# Lifestyle Medicine for Obesity in the Era of Highly Effective Anti-Obesity Treatment

**DOI:** 10.3390/nu17142382

**Published:** 2025-07-21

**Authors:** Deepa Sannidhi, Ruth Abeles, William Andrew, Jonathan P. Bonnet, Kenneth Vitale, Varalakshmi Niranjan, Mahima Gulati, Kaitlyn Pauly, Ryan Moran, Lydia Alexander, Cassidy Le, Suraj Rajan, Camila Romero

**Affiliations:** 1 Department of Family Medicine, UC San Diego School of Medicine, La Jolla, CA 92093, USA; 2 Department of Internal Medicine, UC San Diego School of Medicine, La Jolla, CA 92093, USA; 3 Department of Medicine, School of Medicine, Stanford University, Stanford, CA 94305, USA; 4 Department of Orthopedics, UC San Diego Health, San Diego, CA 92037, USA; 5 Department of Medicine, University of Connecticut Health Center, Farmington, CT 06030, USA; 6 Department of Practice Management, American College of Lifestyle Medicine, Chesterfield, MO 63006, USA; 7 Department of Clinical Services, Enara Health, San Mateo, CA 94403, USA; 8 School of Public Health, UC San Diego Herbert Wertheim, La Jolla, CA 92093, USA; 9 General Preventive Medicine Residency, UC San Diego/San Diego State University, La Jolla, CA 92093, USA

**Keywords:** lifestyle medicine, obesity, intensive lifestyle change programs, healthy lifestyle, intensive behavior change program, exercise, diet

## Abstract

Despite recent advances in the treatment of obesity, lifestyle medicine remains foundational to the treatment of individuals with obesity, regardless of the modality chosen by the patient with the guidance of the clinician they are working with, including in conjunction with, as appropriate, anti-obesity medications and metabolic surgery. Lifestyle medicine involves the use of diet, exercise, sleep, stress, and other lifestyle modalities in the treatment of disease. Clinicians and health systems should, after a patient-centered discussion with the patient, do their best to ensure access to lifestyle treatments. Gold standard guidelines recommend intensive, multicomponent lifestyle change programs for obesity treatments with evidence-based diet and exercise counseling and established, theoretically driven behavior change components. Clinicians treating obesity should be aware of their own biases, make efforts to reduce stigmatizing experiences in their practice, and address weight stigma in their treatment plans as needed. A variety of dietary patterns can be used to support patients with obesity, and clinicians should make evidence-based but patient-centered recommendations that aim to maximize adherence. Diet and exercise can play an important role in reducing the side effects of treatment and optimizing outcomes in weight loss, attenuating the effects of metabolic adaptation, and weight maintenance. Exercise should be increased gradually to reduce injury with a goal of 200–300 min (approximately 3.3–5 h) of moderate to vigorous intensity exercise per week to maximize weight maintenance effects with exercise prescriptions customized to patients risks. A variety of practice models can be leveraged along with the use of an interdisciplinary team to provide lifestyle medicine care for those with obesity.

## 1. Role of Lifestyle Medicine in the Treatment of Obesity

Obesity is a complex chronic disease, the prevalence of which is increasing rapidly globally. It currently affects approximately 40% of US adults [1] and 16% of adults worldwide [2]. Meanwhile, in 2024, the NCD Risk Factor Collaboration (NCD-RisC) [3] published findings which estimate that more than one billion people in the world are now living with obesity, (nearly 880 million adults and 159 million children and adolescents aged 5–19 years). In layman’s terms, one in eight people in the world now suffer from obesity. Projecting into the future, one analysis suggested with high predictive accuracy that by 2030 nearly one in two US adults (48.9%) will have obesity, and almost one in four US adults (24.2%) will have severe obesity. Furthermore, there are significant inequities in the populations affected by this illness. Severe obesity is likely to become the most common BMI category among US adult women (27.6%), non-Hispanic US black adults (31.7%), and low-income US adults (31.7%).

A recent international, multi-stakeholder consensus statement [4] defined clinical obesity as a chronic, systemic illness characterized by alterations in the function of tissues, organs, the entire individual, or a combination thereof, due to excess adiposity. Adiposopathy is defined as adipocyte/adipose tissue dysfunction caused by a positive caloric balance and sedentary lifestyle in genetically and environmentally susceptible individuals [5]. Obesity pathogenesis involves the resetting of the body weight “set point” at an increased value, and has complex multifactorial causation, including the interaction of genetic, epigenetic, and developmental factors with extrinsic, environmental factors (“exposome”) such as exposure to hyperpalatable, energy-dense foods, chemical exposures (“obesogen theory”), and social determinants such as income, built neighborhoods, gender, etc. [6].

Lifestyle medicine (LM) is defined as “a medical specialty that uses therapeutic lifestyle interventions as a primary modality to treat chronic conditions including, but not limited to, cardiovascular diseases, type 2 diabetes, and obesity”. Lifestyle medicine certified clinicians are trained to apply evidence-based, whole-person, prescriptive lifestyle change to prevent, treat, and, when used intensively, often reverse such conditions [7]. The six pillars of LM are a whole-food, plant-predominant eating pattern, physical activity, restorative sleep, stress management, an avoidance of risky substances, and positive social connections [8].

The role of LM in the treatment of obesity focuses on addressing the root causes of obesity through sustainable, evidence-based interventions that emphasize long-term behavioral changes. Lifestyle medicine aims to not only reduce excess weight but also improve overall well-being and prevent obesity-related comorbidities. Achieving sustainable behavioral change to maintain weight loss requires a multidisciplinary approach [9]. LM uses structured lifestyle interventions to address the root causes of obesity and its comorbidities through sustainable, patient-centered care [10]. Based on gold standard recommendations, these interventions are most effective when delivered through intensive, multicomponent programs that integrate nutrition therapy, physical activity, behavioral counseling, and team-based support [11,12]. Yet, challenges such as insurance coverage, time constraints, and health equity gaps remain significant barriers to access [11].

This narrative review is intended for primary care clinicians, lifestyle medicine practitioners, and obesity specialists who seek practical, evidence-based guidance for implementing LM alongside pharmacologic and surgical interventions. We aim to provide a clinically actionable framework grounded in current guidelines, with a focus on nutrition, exercise, behavior change, sleep, stress, and stigma reduction utilizing a multi-disciplinary approach. We explore the evolving role of LM in the era of highly effective obesity treatments, highlight the need for interdisciplinary models of care, and provide practical tools—including summary tables and patient-centered strategies—to optimize outcomes for diverse patient populations. Rarer causes of obesity such as monogenic obesity disorders, syndromic obesity, and hypothalamic obesity are out of the scope of this expert review.

## 2. Overview of Lifestyle Intervention for Treatment of Obesity

Recommendations from major medical societies suggest that lifestyle medicine is the foundation of obesity treatment [12,13,14,15]. More than a particular nutrient composition, studies have made it clear that increased frequency and adherence to counseling sessions is an important factor [16,17,18]. Multiple United States Preventive Services Task Force (USPSTF) recommendation statements recommend intensive lifestyle or intensive behavioral intervention for the treatment of obesity or obesity-related comorbidities [19,20,21,22,23]. In recent years, the use of this evidence as a justification for insurance companies to deny access to obesity treatments has generated discussion [11,24]. This can disproportionately affect patients for whom both obesity treatment and lifestyle medicine is all the more difficult to access [11]. Intensive lifestyle intervention is defined by the USPSTF as at least 12 or more sessions in the first year, with many lasting 1–2 years, with a significant number having a core phase and maintenance phase [19]. Programs should be multicomponent, incorporating diet, exercise, behavior change principles, and motivational interviewing. These interventions may be offered in an individual, group, or mixed format [25]. Although intensive lifestyle intervention is covered by Medicare and multiple insurance companies, which we will discuss further in the section on clinical practice models, it can be hard to access for many patients. In addition to this, individuals with high SES burden, or those whose care is affected by other social determinants of health such as a lack of transportation, housing instability, long work hours, or caregiving responsibilities, or those managing multiple chronic medical problems, may have difficulty adhering to the time commitment necessary for intensive lifestyle intervention [11,24]. In these situations, patients can be referred to a nutritionist for fewer education sessions, or join an asynchronous program involving the use of technology to provide lifestyle support. Additionally, clinicians can offer intensive lifestyle intervention in the office through a variety of billing mechanisms that are discussed in a later section on practice models that support lifestyle medicine approaches including intensive behavioral therapy, shared medical appointments, and others.

The treatment of obesity should be tailored based on the patient’s individual preferences, values, social circumstances, medical comorbidities, and stage and severity of obesity [13,26]. While the clinician can provide a strong recommendation for lifestyle intervention, sharing the risks and benefits, ultimately it is a shared medical decision as to whether the patent is ready or willing to attempt lifestyle behavior modifications, and withholding guideline-directed therapy before pharmacologic or surgical treatments is discouraged, particularly given limitations to access, burden of time, and transportation concerns related to participation in intensive lifestyle interventions. Clinically significant weight loss with lifestyle intervention alone is 5–10% of initial body weight. Large studies including RCTs, on average, show 4–6% weight loss [27]. Patients requiring more weight loss to address the sequelae of obesity may need medications or surgery [27]. If patients have already attempted lifestyle change and have been unsuccessful or desire to lose more weight to improve their quality of life, medications and/or surgery are options and their risks and benefits should be discussed. Individuals with obesity may reach a metabolic “set point” that has been altered over the course of their weight gain [28]. This is what can cause the weight regain experienced by as many as two thirds of participants in dietary interventions for weight loss [29,30]. This is compounded by increases in hunger hormones and the lowering of satiety hormones [28]. The metabolic set point is still a subject for research with cellular, metabolic, neuroendocrine [31], lifestyle, and socio-environmental determinants [32]. If patients have reached a plateau and still desire to lose weight, continued advice to only improve diet and exercise can stigmatize them and may not improve outcomes. Rather, a patient-centered discussion should be had with the patient regarding next steps based on options available.

## 3. Lifestyle Medicine and Obesity Medicine History

An important consideration for clinicians is that many patients with obesity have tried lifestyle intervention before. A significant number have been on a cycle of “yo-yo dieting” since childhood, and continued weight cycling may pose risks [33]. Thorough history taking can elucidate prior lifestyle attempts including what has worked before and what has not, and shared decision making with the patient guided by their personal values, preferences and individual needs can help clinicians suggest an appropriate lifestyle medicine program for patients. We recommend that patients be asked about prior weight loss attempts, eating triggers and habits, and the amount of weekly physical activity. A detailed list of historical elements is listed in Table 1.

In addition, a 24 h or 3-day diet recall or 3 day food diary may be helpful for dietary assessment in addition to a food frequency questionnaire (examples given in Table 1). Assessing diet from a variety of approaches can help to identify problematic eating behaviors given that people consistently under-report caloric intake in a variety of assessment methods [34].

**Table 1:** Obesity and Lifestyle Medicine Assessment.

Assessment Area	Elements of Assessment/Assessment Tools
Weight trajectory	- Age at onset: “How old were you when you first started struggling with your weight?” - Triggers for weight gain: job changes, injuries, medical conditions, medications (steroids or anti-psychotics), puberty, pregnancy, and menopause
Previous weight loss attempts	- Previous diets approaches and programs, and dietary patterns tried in the past (e.g., low carb, low fat, intermittent fasting, or calorie/macronutrient tracking) - Medically supervised programs - Structured group or individual programs - Medications and supplements - Surgeries and devices - Meal replacements (e.g., shakes or pre-made meals). - Assess factors linked to adherence: “What worked well for you? What did not work well for you? If something worked well, what caused you to stop using the approach?” - History of eating disorders: o Bingeing, purging, and restricting; o Hospitalizations for eating disorders; o Date of remission; o Number of relapses and weight loss attempts since remission from eating disorder. - Night eating (eating late at night or waking up to eat)
Eating behaviors	- Eating triggers: people, places, activities, and emotions (happiness, sadness, anger, stress, or boredom) - Meal patterns: home-cooked vs. restaurant meals, social events, late-night eating, convenience foods, and grazing - High-risk foods such as sugar-sweetened beverages, alcohol, savory snacks, and sugary foods - Distracted/mindless eating (e.g., in front of screens or while multitasking) - Three-day food diary, 24 h or 3-day diet recall, and/or food frequency questionnaires - In-office diet screeners include the MEDAS (Mediterranean Diet Adherence Screener [35]), REAP and WAVE (Rapid Eating Assessment for Participants and the corresponding shortened questionnaire and Weight, Activity, Variety, and Excess) [36], Mini-EAT (Eating Assessment Tool) [37], the rPDQS (Rapid Prime Dietary Quality Screener) [38], and the ACLM (American College of Lifestyle Medicine) diet screener [39]
Exercise	- Ask about frequency and duration and quantify total in hours or minutes per week. E.g., two Zumba classes an hour each and three walks per week for 30 min = 210 min per week

## 4. Role of Other Lifestyle Medicine Pillars

A thorough lifestyle history including an assessment of sleep, stress levels, substance use, and social connectedness and support is especially important when applying prescriptive lifestyle change to prevent, treat, and, when used intensively, potentially reverse obesity. Screening for obstructive sleep apnea (OSA) is important for individuals with obesity, and an immediate sleep study referral is recommended, if they are not already diagnosed and being treated. There is a dose–response relationship between obesity and OSA, with more weight loss leading to decreases in OSA severity, as seen in a recent RCT where weight loss (≥10%) led to approximately a 50% decline in median AHI and a >60% reduction in OSA-related symptoms [40]. Additionally, screening for other sleep disturbances such as shift work and insomnia is important due to the adverse impact poor quality sleep has on weight. This is elucidated in the Wisconsin sleep cohort study where reductions in slow-wave (N3) and REM sleep, which are key components of restorative sleep, are associated with weight gain and obesity [40]. Patients with insomnia can be provided education about sleep hygiene [41] and are referred to therapists specializing in CBT for insomnia (CBTi) [42]. In the absence of access to CBTi, resources from the Veterans Administration such as the CBTi coach app.

Alcohol use can be a common contributor to obesity and can exacerbate metabolic abnormalities as well as put patients with obesity at an increased risk of a progression of metabolically associated steatotic liver disease [43]. Tobacco use should be screened for in patients with obesity. Tobacco cessation can ironically cause weight gain for some patients and tobacco cessation efforts may warrant closer monitoring and counseling as patients with obesity may be at higher risk for weight gain/regain.

Stress levels or mental health disturbances such as generalized anxiety disorder or depression may be a frequent contributor to off-plan eating [31]. Such patients can be referred to mental health resources or provided resources for stress reduction practices. Patients may be asked about what they are currently doing for their stress and clinicians can work with them to harness already existing resources such as diaphragmatic breathing practices, guided imagery, progressive muscle relaxation, hobbies, their social support network, or spiritual practices. Lifestyle inventories exist such as the Lifestyle Medicine Health Behavior Scale [44] and the Lifestyle Medicine Inventory [45]. Other practical tools based on validated questionnaires include the 10-item Lifestyle Vital Signs Questionnaire [46] and the American College of Lifestyle Medicine/Loma Linda University short- and long-form lifestyle medicine assessments, which are also available in French and Spanish at https://lifestylemedicine.org/tools-and-resources/ (accessed on 29 June 2025). After assessing the patient’s stress, toxic substance use, social support, and mental health conditions, having a multidisciplinary team or referral network in place so that the appropriate referrals and/or warm hand-offs occur in a timely manner. See Table 1 and Table 2 for a summary of the lifestyle-related screening recommendations.

## 5. Characteristics of Behavioral and Lifestyle Interventions

In accordance with guidelines, where possible, individuals undergoing treatment for obesity should be provided intensive multicomponent interventions. The programs do not need to be conducted by a physician but should be multicomponent in nature, providing information about diet, exercise, and behavioral approaches to weight loss. See Table 3 for detailed information on characteristics of intensive multicomponent lifestyle change programs [19,47] based on the research to date as well as recommendations from the USPSTF. The most widely known of these programs is the Diabetes Prevention Program (DPP), which is based on a multi-site randomized controlled trial among patients with overweight or obesity who had prediabetes that showed a 58% risk reduction of developing type 2 diabetes over a 2.8 year period [48]. The entire curriculum, accredited by the CDC, is free and available on the CDC’s website. Providers can direct the patient to find a program on its website [48].

Intensive behavioral therapy, as defined by CMS, is an intensive program that can be provided by primary care physicians or their staff incident to the physicians, and includes 14–15 sessions [49]. Intensive lifestyle intervention or intensive behavioral therapy typically includes information about diet and exercise to increase patient knowledge and skills, and also includes information about behavioral aspects of weight management which include cognitive restructuring, social support, and goal setting, to name a few, and are detailed in Table 3 [25]. Resources exist for providing intensive behavioral therapy, such as the University of Pennsylvania Model IBT program and the Prevent T2 curriculum for the CDC Diabetes Prevention Program. Table 3 contains links to freely available IBT curricula [48]. Characteristics of behavior change programs that support weight loss and positive outcomes include the use of motivational interviewing and acceptance and commitment therapy over providing conventional advice [50], and the use of other established behavior change techniques detailed in Table 3. Programs should engage in a systematic, theoretically driven evaluation of the facilitators and barriers to behavior change in the social and organizational context and have a strong focus on weight maintenance [25]. In order to increase access for those with transportation and time limitations, remote interventions delivered in an intensive fashion by telephone or video conference have shown some benefit [50].

**Table 3:** Characteristics of Intensive Multi-component Lifestyle Interventions.

	Key Characteristics of Intensive Multicomponent Lifestyle Interventions [25,51]
Program Structure	**≥8 sessions over 12 weeks or ≥12 sessions per year** Often last **6 months to 1 year** Delivered by physicians or trained non-physician staff Must include **diet, physical activity, and behavioral strategies** [12,52]
Examples of Established Programs	**Diabetes Prevention Program (DPP)**: - Based on a large RCT, showed **58% reduction in T2D risk** over 2.8 years for overweight/obese individuals with prediabetes; **CDC-accredited curriculum** is free and publicly available; “Find a Program” tool on the CDC website can be used for referrals [48,53] **Model-IBT program for providing intensive behavioral therapy in** primary care settings - Modeled on the DPP to meet CMS requirements for the provision of IBT [51]
Behavioral Components [25,51,52]	**Cognitive restructuring** (e.g., managing black-and-white thinking) Handling food triggers and social situations **Problem-solving** around off-plan eating **Self-monitoring**, **stimulus control**, and **relapse prevention** **Self-assertion**, **goal setting**, **social support**, **self-reinforcement**, **barrier identification**, **coping skills**, **contingency management**, and **action planning** Use **motivational interviewing** rather than directive advice, respecting the patient’s autonomy Employ **established behavior change techniques** Instruction on **healthy diet and physical activity** **Self-monitoring** and relapse prevention Use of **pedometers**, **step goals**, and physical activity diaries **Tailored physical activity** guidance Mobilize **social support** Emphasis on **weight maintenance**
Evaluation Strategy [25,51,52]	Use a **theoretically grounded, systematic approach** Assess **barriers and facilitators** within **social and organizational context**

## 6. The Effect of Weight Stigma in Healthcare and Its Impact on the Treatment of Obesity

One cannot discuss behavioral approaches to addressing obesity without addressing weight stigma. Weight stigma is a term to describe the thoughts and acts of discrimination toward individuals due to their weight and size [54]. Weight stigma has been linked to lower morbidity and mortality. There is significant evidence that indicates these judgements and behaviors can lead to decreased access to care, delay in seeking care, and strained patient–provider relationships, all of which contribute to poor medical outcomes. [55]. Additionally, the literature has repeatedly demonstrated that negative self-image, internalized weight bias, and experiencing implicit and explicit bias lead to weight gain and binge-eating behaviors [56]. These effects are not isolated to Western societies and many of them have been demonstrated in studies in several countries [57,58]. While healthcare providers are not acting out of a conscious desire to stigmatize patients with obesity, they often perpetuate weight stigma through less conscious behaviors such as the moralization of weight status, avoiding eye contact, physical touch, providing overly simplified or unsolicited weight loss advice, or not having appropriately sized equipment available for patients such as chairs, blood pressure cuffs, and gowns [54,56]. We recommend healthcare providers be proactive in providing patients with education that helps them to understand the multi-causal nature of obesity with genetic and socio-environmental determinants. Decorating offices with art depicting people of different sizes can be helpful, as well as using marketing materials for practices with non-stigmatizing pictures of people of various sizes. In recent years, mindful self-compassion-based interventions have provided some promising evidence for helping patients with weight stigma and internalized weight bias [59,60]. Referral to cognitive therapy and psychotherapy support for patients with significant internalized weight bias is best practice for improved response to pharmacotherapy and surgery [54].

Clinicians should avoid using terms that have been shown to stigmatize patients with obesity such as “fat” or “obese”, as well as language that is blaming or insensitive. The focus should be on using motivational interviewing approaches that respect patient autonomy, and respect patient decisions about body weight, whether weight loss is a goal for patients or not. Most importantly, healthcare providers must recognize their own conscious or implicit biases, which once identified can be addressed with compassion training. Once these barriers have been removed, a provider can more honestly engage with their patients and move towards exploring their personal experiences and hesitations, if any [42].

This has implications for how lifestyle advice is provided to patients as well. There is evidence that people with obesity self-exclude from sports due to stigma [61]. People who experience weight stigma and internalized weight bias have higher motivation to avoid exercise and decreased self-efficacy to engage in exercise. There is little evidence to guide clinicians in their counseling on physical activity in a way that reduces stigma, though there are some frameworks emerging that can guide further research [62]. Much of this comes from the Health At Every Size (HAES) movement and research on body appreciation and self-compassion [63]. Appearance-oriented motivation for exercise may mediate an association between body surveillance (monitoring appearance and concern with other’s perceptions of one’s appearance) and exercise avoidance [64]. Conversely, body appreciation mediates a relationship between self-compassion and an intrinsic motivation for physical activity [65]. Thus, focusing on weight-inclusive, body-positive messaging may improve exercise intention as well as fitness/health motivation while promoting adaptive exercise behaviors [66]. Another approach includes creating safe spaces for group exercise that focus on a cultural and leadership commitment to inclusion, physical activity spaces that are adapted for larger bodies with fewer mirrors and large windows, the removal of body-weight-specific messaging, fostering positive community and a sense of belonging, and increased motivation [62]. Further benefits of providing a safe space for group exercise include a supportive environment balancing encouragement with accountability but can also include improved exercise prescription adherence and consistency in maintaining physical activity.

The experience of the authors has been that it can be helpful to use inclusive language like “movement” and “activity”. This inclusive terminology also helps broaden the approach to exercise to encourage intrinsic motivations like well-being, and includes any activity patients may enjoy, including increasing non-exercise activity thermogenesis (NEAT) as almost any movement has been shown to have positive health benefits in a dose-dependent manner. The CDC’s physical activity guidelines recommend creating specific, action-oriented goals [54]. It is helpful to have these goals be patient-driven, and for the clinician to withhold personal biases about what the patient’s fitness level is (such as assuming someone with obesity is a “beginner”) while also screening to prevent injury. For those interested in learning more, the Body Positive Fitness Alliance and the American Council on Exercise resources on weight inclusivity can be helpful.

Similarly to physical activity, it is important not to assume that patients have not tried to lose weight using diet changes in the past. Focusing on behaviorally centered, process-oriented goals instead of weight-loss goals can be helpful. Help patients make the connection between the health benefits of their behaviors regardless of their weight and as above, respect patients’ decisions about how to handle their weight. For example, the Diabetes Prevention Program outcomes study (DPPOS) showed protection from progression to diabetes despite weight regain [55]. Intuitive eating or similar models such as the EC Satter model or a “weight neutral” approach to healthy behavior can be a helpful approach for patients who wish to focus on their health but not their weight. While weight loss may or may not be a result of such approaches, they can prevent weight cycling [67,68] and have independent health benefits [69,70]. Volumetrics or approaches that focus on the calorie density of foods can also improve health and weight-related outcomes while allowing individuals on those diets to eat to satiation [71,72].

For patients that express a desire to lose weight, the authors suggest incorporating some form of self-monitoring of food and/or weight based on patient preferences and confidence level to execute such behaviors, while also providing resources to assist the patient in doing so. While evidence-based weight loss interventions typically incorporate some form of self-monitoring, eating disorders and disordered eating are prevalent in those with obesity, and many individuals with obesity may have engaged in unhealthful weight control behaviors in their youth and young adulthood [73,74], so it is important to provide such recommendations in a patient-centered manner in real-world practice, and after screening for eating disorders.

It can be important for obesity medicine physicians to have a multi-disciplinary referral network of dietitians, fitness specialists, psychiatrists, and psychologists who specialize in eating disorders or weight-inclusive approaches.

## 7. The Role of Lifestyle Medicine in the Era of GLP-1 RA and Other Anti-Obesity Medications

In recent years, prescriptions for highly effective obesity medications (OMs) have increased significantly, particularly GLP-1 receptor agonists (GLP-1 RA). Since its approval for diabetes, and subsequently for obesity, physicians have become much more comfortable prescribing semaglutide [75]. One Danish study showed 86% of physicians reported prescribing semaglutide [76]. While only a minority of those eligible are receiving obesity pharmacotherapy, a growing numbers of primary care physicians and advanced practitioners are now comfortable prescribing obesity medication [77]. Driving some of this is the efficacy of the medications, with semaglutide showing ~14% weight loss in clinical trials, and tirzepatide demonstrating ~20% weight loss in clinical trials [78,79]. In the landmark studies that led to FDA approval for these medications, lifestyle intervention led to only marginal improvements in weight loss outcomes [78,79,80]. While this may lead some to conjecture on the utility of lifestyle intervention, such a conclusion would be premature.

Best practice guidelines recommend lifestyle intervention in conjunction with the treatment of obesity. Additionally, discontinuation rates for GLP-1 RAs can be very high for a variety of reasons [81]. Both semaglutide and tirzepatide were FDA approved to be provided in conjunction with a reduced calorie diet and exercise [82,83]. Therefore, it is the position of the authors that the best practice for medical providers, health systems, and insurance companies is to provide patients access to intensive, multicomponent obesity treatment based on patient-centered decision making [14,53]. There is some evidence for improvement in outcome for GLP-1 RAs when combined with lifestyle change, such as in the MODEL-IBT study, in which liraglutide combined with intensive behavioral therapy produced close to 12% weight loss [51]. This is in contrast to clinical trials of liraglutide, where the average weight loss is 6–8% [84]. Additionally, the SURMOUNT-3 study showed weight loss of 25% of initial body weight after receiving tirzepatide in patients who had already achieved greater than or equal to 5% weight loss with intensive lifestyle intervention [85]. This suggests that additional benefit may be derived from pairing GLP-1 RA medication with intensive lifestyle intervention.

### 7.1. Nutrition Recommendations for Those on Anti-Obesity Medications

Lifestyle intervention can also reduce side effects in patients taking GLP-1 receptor agonists including recommending smaller (and if needed, more frequent) meals that are lower in fat [14,86]. This recommendation also applies to patients taking orlistat, the side effects of which result from fat malabsorption [87].

GLP-1 RAs have been linked to significant muscle mass loss, though the clinical significance of this loss is unclear, particularly given no significant change in relative lean mass, and some improvements in muscle quality [88,89,90]. Nevertheless, muscle loss may be a concern particularly for patients at risk of sarcopenia, particularly the frail or elderly. [90]. Adequate protein intake and strength training have been shown to mitigate muscle loss linked to GLP-1 RAs, which is discussed in the sections on nutrition and exercise below [90,91,92]. Rapid weight loss including from GLP-1 RAs can also cause muscle and bone loss, and can potentially contribute to weight regain [93,94,95]. Therefore, slower weight loss through a slower titration of medication may be considered as well, based on timing considerations (e.g., patients may be trying to lose weight for a needed surgery).

Counseling patients on adequate nutrition can be helpful in preventing nutrient deficiencies such as vitamins D, A, B6, folate, C, calcium, iron, magnesium, potassium, and zinc, which can be common in people with obesity and for those on hypocaloric diets [96,97,98,99]. Individuals with obesity on GLP-1 RAs may have some difficulty ensuring adequate protein intake. A more detailed discussion of protein is outlined in the section on dietary principles for obesity treatment.

Fiber intake can reduce constipation, and though there is not adequate evidence of this in GLP-1 RAs, it is the authors’ experience that higher fiber foods can be helpful [100]. Fiber supplements such as psyllium and wheat dextran can also assist with diarrhea resulting from GLP-1 RAs, bupropion, and naltrexone, as well as phentermine. While fiber is a nutrient of concern and intake should be encouraged, higher fiber intake can sometimes exacerbate side effects [101]. There are few guidelines and studies to guide practice. Beans, cruciferous vegetables and other high fodmap foods that are more prone to gas formation may be reduced in the diet and can be reintroduced slowly if patients are experiencing bloating or cramping similar to IBS [102]. One may consider using the University of Monash guides for FODMAP restriction and reintroduction.

### 7.2. Physical Activity Recommendations for Patients on AOMs

Physical activity recommendations may change based on the medication being used to treat the patient’s obesity. In general, increased vigilance is recommended during periods of medication initiation and dose titration as this is the time when medication effects will be the most apparent and have the most individual variability. Common side effects of anti-obesity medications are abdominal pain, diarrhea, and nausea [87,103,104]. These are best managed with appropriate dose titration, or timing the medication appropriately (as with bupropion/naltrexone), though activities that may exacerbate these symptoms, such as extremely high-intensity exercise, may be initially avoided. If digestive side effects become severe, while symptomatic management and perhaps medication discontinuation may be considered, in the interim, the side effects may necessitate adjustments in the timing, place, and type of physical activity to avoid discomfort during exercise [87]. Often, patients on any obesity medication, but particularly incretin medications, are at risk for a low oral intake of fluids and solid food, predisposing to dehydration, and patients, particularly those engaging in vigorous or intense physical activity, should be encouraged to hydrate and eat water-rich foods [104,105].

Additional considerations for some medications involve associations with hypotension, hypoglycemia, (incretins), and CNS depression, all of which warrant safety precautions for any altered level of consciousness such as avoiding exercise alone and taking falls precautions [87,104]. Dizziness and ocular side effects accompanying these side effects, as with topiramate, may necessitate falls precautions as well [106]. This is particularly the case for activities involving aquatics, e.g., swimming, surfing, and boating, as this can increase the risk of drowning events, as well as activities such as cycling. Activities involving balance, e.g., projectile sports, gymnastics, and even advanced yoga techniques, warrant careful consideration. Some medications (topiramate) can be associated with hypohydrosis and heat intolerance [106]. Environmental considerations need to be taken to reduce the risk of heat injury [87,106].

Stimulants such as phentermine and bupropion are associated with hypertension, tachycardia, and arrhythmias and patients should be screened for a personal and family history of cardiac disease and a personal history of hyperthyroidism, psychosis, glaucoma, [87,103], and substance use disorders (phentermine). In patients without these risk factors, there are no known contraindications to exercise, particularly cardiovascular activity but also high-level resistance training. However, the careful monitoring of any cardiovascular symptoms is recommended, and slow titration of both the medication and physical activity should be encouraged [87]. These medications may require the monitoring of blood pressure and adjustments in exercise intensity to ensure patient safety [87].

Utilizing pharmacists to assist with medication management for obesity, provide lifestyle advice, and provide counsel regarding side effects and dose adjustments could be a beneficial approach [107]. Table 4 summarizes lifestyle considerations for commonly used anti-obesity medications.

## 8. Role of Lifestyle Medicine in the Care of Metabolic and Bariatric Surgery Patients

Bariatric surgery is indicated for patients with a BMI of 40 or greater, or a BMI of 35 or greater with at least one serious obesity-related comorbidity, according to the American Association of Clinical Endocrinologists, The Obesity Society, and the American Society for Metabolic and Bariatric Surgery [12,15,109]. Emerging evidence also supports considering bariatric surgery for patients with type 2 diabetes and a BMI of 30 to 35 if metabolic conditions are inadequately controlled despite optimal medical treatment [109,110].

Bariatric surgery is a powerful metabolic intervention. It leads to significant and sustained weight loss, with improvements in obesity-related comorbidities such as type 2 diabetes, hypertension, dyslipidemia, and obstructive sleep apnea [110]. Long-term studies have shown that bariatric surgery is associated with a lower incidence of cardiovascular events, cancer, and mortality [111,112]. Bariatric surgery has also been shown to reduce the risk of severe chronic kidney disease, coronary disease, diabetes, inflammatory bowel disease, hypertension, chronic pulmonary disease, asthma, cancer, and chronic heart failure [110,111,112]. However, clinicians must be aware that bariatric surgery is associated with an increased risk of hospitalization and additional surgeries, as well as certain morbidities such as peptic ulcers, alcohol misuse, frailty, severe constipation, sleep disturbance, depression, and chronic pain.

Lifestyle interventions provided by a multidisciplinary team pre- and post-surgery play a major role in the outcomes of bariatric surgery. Preoperative weight loss using lifestyle interventions by a supervised weight-management program is usually recommended [113]. Studies have shown patients with modest preoperative weight loss have surgical advantages, such as a shortened surgery time and decreased postoperative complications [113,114]. The 2019 updated guidelines by the American Association of Clinical Endocrinologists/American College of Endocrinology, The Obesity Society, the American Society for Metabolic & Bariatric Surgery, the Obesity Medicine Association, and the American Society of Anesthesiologists recommend comprehensive lifestyle medicine evaluation including a healthy eating index, cardiovascular training, strength training, sleep hygiene, mood and happiness, alcohol and tobacco use, substance use, and community engagement in addition to assessment by a dietitian and a psychologist. They also recommend referral for psychological counseling and smoking cessation counseling as well continuance of perioperative weight loss efforts [115].

The American College of Surgeons Metabolic and Bariatric Surgery Accreditation and Quality Improvement Program (MBSAQIP) is dedicated to enhancing the safety and quality of care for bariatric patients in the United States and Canada. Their standards recommend that prior to surgery, bariatric centers offer a nutritional assessment by a registered dietitian with expertise in metabolic bariatric surgery (MBS) [116]. Dieticians help obtain a comprehensive weight history, identify maladaptive eating behaviors or patterns, correct any micronutrient deficiencies prior to surgery as well as provide preoperative nutrition education. All patients should be encouraged to participate in ongoing support groups after discharge from the hospital. In those patients in whom T2DM, hyperlipidemia, and hypertension have resolved, continued surveillance is recommended [115].

Lifestyle medicine approaches, including diet and physical activity, also play an important role in the immediate post-surgical period as well as in the maintenance of weight loss after bariatric surgery [115]. A registered dietitian with expertise in MBS can assist in the management of postoperative patients who may be experiencing food intolerances, malabsorption issues and micronutrient deficiencies, and weight regain [109]. The Obesity Society and American Society for Metabolic & Bariatric Surgery guidelines for the perioperative nutritional, metabolic support of the bariatric surgery patients recommend lifelong routine metabolic and nutritional monitoring after all bariatric surgical procedures [53,112].

Common factors associated with weight regain after bariatric surgery include anxiety, sweet consumption, emotional eating, portion size, food urges, binge eating, and a loss of control/disinhibition when eating. Referral to a behavioral health provider may be helpful in addressing these behaviors [117]. In the long term, avoiding nutrient deficiency and weight regain is achieved by patient education with meal prep and meal planning, and appropriate micronutrient supplementation [118]. While it is not clear that particular nutrients need to be consumed at higher levels, evidence suggests at least 60 g of protein and up to 1.5 g of protein per kg of ideal body weight to maintain muscle mass. Higher protein intakes of 108 g or more can assist with increasing muscle strength [119]. A more detailed discussion of protein is provided later in this article in the section on diet quality. Another long-term physiological consideration involving malabsorption includes iron and vitamin B12. Deficiencies in these nutrients can lead to anemia which can manifest as fatigue, decreased exercise capacity, and even lightheadedness. Similar fall precautions should be taken in those who may be at high risk, e.g., difficulty adhering to postoperative nutrition plans, food insecurity, etc. Patients with a history of bariatric surgery should be counseled to also increase their fluid intake [119].

According to the American Association of Clinical Endocrinologists, patients are advised to increase their physical activity (aerobic and strength training) to a minimum of 30 min per day as well as increase physical activity throughout the day as tolerated. The benefits of physical activity in relation to bariatric surgery are further discussed in the section on physical activity in obesity treatment later in this article.

Different levels of physical activity and precautions may be recommended at different points in the postoperative course. Early postoperative considerations within the first 30 days of surgery can include nausea, vomiting, dehydration, surgical site infection, cardiac dysrhythmias, venous thromboembolisms, atelectasis, and pneumonia. Generally, activity is limited to gentle activities of daily living and low volume and intensity walking [115]. Later complications for which clinicians should maintain a high level of suspicion can involve internal complications such as fistulas, band erosions, hernias, strictures, and bowel obstructions. There are no known risks with exercise or specific activity restrictions during this period [115,120]. A complication unique to bariatric surgeries that utilize bypass techniques is dumping syndrome which can be triggered by high glycemic index foods, most common within the first 18 months of surgery. This results in facial flushing, lightheadedness, fatigue, reactive hypoglycemia, and postprandial diarrhea [121]. In these patients, it may be useful to exercise caution with activity shortly after mealtimes and to consider safety precautions such as avoiding aquatic activities, exercise involving high risk related to falls, and unsupervised/solo activity especially in areas with environmental exposure risk.

Patients with a history of bariatric surgery may also be at increased risk of osteoporosis. While society guidelines focus on weight-bearing and resistance exercise to prevent osteoporosis [26], any type of exercise provides an additional benefit in these patients to maintain bone mineral density [122,123]. Clinicians should have a low threshold to evaluate for bony stress injuries or fractures should any pain out of proportion or atypical musculoskeletal issues arise for patients engaging in high-energy, repetitive impact exercise. Table 5 summarizes lifestyle strategies for optimizing surgical outcomes pre-, peri- and post-operatively.

## 9. The Role of Diet in the Treatment of Obesity

Lifestyle change, including dietary adjustments, is a central part of effective obesity treatment plans. Weight loss is induced by maintaining an energy deficit through the reduction of caloric intake, an increase in energy expenditure through physical activity, or, ideally, a combination of both. A daily caloric deficit of approximately 500 to 750 calories is a common target for weight loss interventions, with most women aiming for 1200–1500 kcal/day and most men aiming for 1500–1800 kcal/day, adjusted for individual baseline body weight [124]. Another approach is to estimate the basic metabolic rate.

Accurately estimating the basal metabolic rate (BMR) is essential for tailoring caloric intake in obesity treatment. The Mifflin–St Jeor equation is one of the most validated and widely used formulas:

- **Men**: BMR = 10 × weight (kg) + 6.25 × height (cm) − 5 × age (years) + 5.
- **Women**: BMR = 10 × weight (kg) + 6.25 × height (cm) − 5 × age (years) − 161.

Total daily energy expenditure (TDEE) is then estimated by multiplying the BMR by an activity factor. This framework supports energy deficit planning for weight loss. A recent study by Karagun and Baklaci confirmed that the Mifflin–St Jeor equation closely approximates the BMR compared to indirect calorimetry in individuals with overweight and obesity [125]. For practical use, tools such as the national academy of sports medicine (NASM) calorie calculator (https://www.nasm.org/resources/calorie-calculator, accessed on 5 July 2025) or the NIH body weight planner (https://www.niddk.nih.gov/bwp accessed on 5 July 2025) can be used to calculate calorie needs and calorie deficits.

Another clinical consideration when recommending caloric restriction is the feasibility and effect of self-monitoring and self-weighing on patients. Caloric restriction warrants tracking calories and portions through apps and other means, which has been linked to disordered eating behaviors and negative body image in some studies [126,127]. Long-term adherence to self-monitoring has been linked to habit formation, so using routinization, cues, implementation intentions, action planning, goal setting, and context-dependent repetition may improve adherence [128,129].

Those with obesity who have engaged in some form of calorie or dietary restriction have a higher likelihood of developing nutritional deficiencies [53,98,99,130,131], especially patients who have pre-existing conditions that may limit or interrupt the absorption of nutrients (i.e., inflammatory bowel disease [132], a history of bariatric surgery, etc. [133,134]). Even with supplement use, gaps persist. A 2024 study evaluating supplement users across multiple dietary patterns—including vegan, vegetarian, and low-carbohydrate high-fat diets—found persistent inadequacies in vitamin B12 and iron intake, underscoring the need for tailored monitoring [135]. Since dietary restriction or inflammatory bowel disease or surgery can unmask latent deficiencies, in primary care, clinicians should screen high-risk patients for iron status (serum iron, iron binding capacity, ferritin), B12, 25-hydroxyvitamin D, and other key markers, particularly when dietary restrictions or gastrointestinal comorbidities are present. Collaboration with registered dietitians (RDs) is essential, especially for patients navigating multiple dietary constraints. Choosing a diet that is tailored to the patient’s preferences and takes into account any conditions impacting nutrient absorption or heightened risk is an important part of comprehensive care delivery for patients with obesity.

Calorically restricted high-protein diets are commonly used in weight loss due to protein’s ability to help maintain lean tissue during active weight loss. Protein serves multiple purposes in obesity treatment in that it reduces the risk of lean mass loss while also aiding muscle protein synthesis during exercise [136]. In addition to maintaining lean tissue, protein in the diet has also been found to induce satiety, helping patients feel fuller longer [137]. The USDA recommendation for daily protein intake is 0.8 g/kg per day. Since adipose tissue does not have significant protein needs, this may be calculated based on adjusted or ideal body weight if lean body mass data from DEXA is not available [138]. These recommendations are based on nitrogen balance studies determining the amount of protein intake required to maintain nitrogen balance and it is unclear how much they can translate to actual clinical practice [139]. For the elderly or those engaging in vigorous exercise, those undergoing rapid weight loss, or strength training, this may increase to 1.2 g/kg of adjusted body weight [138,140]. Some literature suggests protein intake of at 60 g per day and up to 1.2–1.6 g per day of adjusted body weight (25–35% of total calorie intake) during weight loss may be beneficial given increased protein needs during weight loss and in various populations at risk for sarcopenia such as older adults, those with type 2 diabetes and post-menopausal women [136,137,138,141,142,143]. Multiple society recommendations suggest higher protein intakes up to 1.4 g–2 g/kg/day provide a benefit of weight maintenance and the maintenance of lean body mass in those engaging in intensive exercise to build muscle and reduce the risk of metabolic adaptation.

The challenges of meal planning, tracking, and GI side effects may create a challenge for adherence [144,145,146]. Protein bars and drinks or meal replacements may be used to supplement in this case, but patients should be advised of the possibility of higher sugar content or the presence of non-caloric sweeteners, which for some patients can lead to increased hunger and have an association with obesity in some studies [147], and may cause gastrointestinal distress for others [148]. It can be helpful for clinicians to have examples of meal replacements and protein supplements with healthy ingredients that they recommend, as well as lists of healthy protein sources minimizing sodium and sugar intake.

The ADA states that high-protein diets providing 20% of total calories, or more than 1.3 g/kg/day are not recommended for patients with chronic kidney disease due to increased risk of albuminuria, more rapid kidney function loss, and increased CVD mortality [149]. Those with chronic kidney disease without diabetes may benefit from a lower protein diet [150] and disease may be accelerated by a higher protein diet, particularly protein from red and processed meat vs. plant-based sources [151]. A summary of protein recommendations is found in Table 6.

Overall it should be noted that lifestyle change interventions provide an expected weight loss between 5 and 10% [17,152] with the potential for significant weight regain [95], though as seen in the diabetes prevention program outcomes study, cardiometabolic benefits may be preserved despite weight regain [153].

**Table 6:** Nutrition recommendations for the treatment of obesity.

**Approaches to caloric deficit for weight loss**	- **Target:** - Caloric restriction using tracking, monitoring, and portion control can exacerbate negative body image and disordered eating [126,127]. - Long-term adherence to self-monitoring has been linked to habit formation, so use routinization, cues, implementation intentions, action planning, goal setting, and context-dependent repetition [128,129]. - 500–750 kcal/day deficit o Women: Aim for 1200–1500 kcal/day. o Men: Aim for 1500–1800 kcal/day (adjusted by baseline weight) [124]. - Consider calculating based on total daily energy expenditure (TDEE) using - TDEE = BMR* activity factor and calculating for a 500–750 kcal deficit [125]. - **Basal metabolic rate (BMR) estimation: Mifflin–St Jeor equation** o Men: 10 × weight (kg) + 6.25 × height (cm) − 5 × age (y) + 5. o Women: 10 × weight (kg) + 6.25 × height (cm) − 5 × age (y) − 161. - **Tools for calorie calculation** o NASM Calorie Calculator: nasm.org/calorie-calculator (https://www.nasm.org/resources/calorie-calculator, accessed on 29 June 2025). o NIH Body Weight Planner: niddk.nih.gov/bwp (https://www.niddk.nih.gov/bwp, accessed on 29 June 2025).
**Screen for nutritional deficiencies**	- Restricting calories or macronutrients can put those with excess weight at increased risk of deficiency [53,98,99,130,131]. - Those with a history of bariatric surgery [133,134], inflammatory bowel disease [132], and dietary restrictions (vegan or low carb) are among individuals who need increased monitoring. - Clinicians should screen high-risk patients for o Ferritin; o Vitamin B12; o 25-hydroxyvitamin D; o Others based on risk factors [135].
**General principles of diet quality**	- High-quality diets emphasize whole/minimally processed foods (fruits, vegetables, cooked whole grains, legumes, nuts, and lean proteins), which are associated with a lower risk of obesity, diabetes, and cardiovascular disease. o These foods are nutrient dense and high in fiber and water, providing low calorie density, which has been shown to allow for a reduction of caloric intake while reducing hunger. o They also support gut microbiota and glycemic control. o Resistant starch and soluble fiber in plant foods can stimulate endogenous GLP-1 production [154,155]. - Avoid ultra-processed foods such as fried foods and foods high in sugars, refined grains, fats, and additives. - Artificial sweeteners have a possible link to obesity and may cause increased hunger, bloating, and diarrhea.
**Reduce calorie density** [72,156]	- Very-low-energy-density foods include most fruit, non-starchy vegetables, and broth-based soups and salads (not counting condiments and dressings) and can be eaten in large portions (less than 0.6 kcal/g). - Low-energy-density foods include whole grains, lean proteins, legumes, and low-fat dairy and can be eaten in normal portions (0.6–1.5 kcal/g). - Control portions of breads, desserts, fat-free baked snacks, full-fat dairy, cheeses, and higher fat meats (1.6–3.9 kcal/g). - Significantly limit portions of fried snacks, candy, cookies, nuts, and fats such as oil, butter, and lard (4–9 kcal/g).
**Recommend healthy protein intake**	- Overall protein intake generally remains stable across diets, with ratios of fats and carbohydrates typically variable [157]. - Helping patients choose high-quality and lean proteins (from whole foods or selected supplements) supports satiety and weight maintenance and prevents muscle loss. - The literature on ideal protein recommendations for weight loss is heterogeneous, with intakes ranging from 0.8 g/kg of ideal body weight to 1.6 g/kg of actual body weight [136,138,142]. - The USDA recommends 0.8 g/kg of body weight. In obesity, since adipose tissue does not utilize protein, this calculation may be based on adjusted or ideal body weight when lean body mass from DEXA or bioimpedance data is not available [138]. - Elderly individuals, those undergoing weight loss, and those who are engaging in an intensive strength training regime may benefit from 1.2 g–1.6 g/kg of adjusted or ideal body weight [136,141,158]. - Higher protein intakes up to 1.4 g–2 g/kg/day provide a benefit for satiety, weight maintenance, and the maintenance of lean body mass in those engaging in intensive exercise to build muscle and reduce the risk of metabolic adaptation [144,145,146]. - Those with chronic kidney disease are not advised to consume protein more than 1.3 g/kg/day or >20% of caloric intake [152]. - Examples of high fiber and lean proteins include legumes such as soy, certain high-protein whole grains such as teff, oats, and quinoa, low-fat dairy, lean meats, mushrooms, seafood, and egg whites [136,144,145,146,159,160]. - When it becomes difficult to include whole food sources of protein, protein supplements such as protein powders, whey protein, and defatted peanut butter powder may be a practical solution. Patients should be steered toward lower sodium versions of these [136,144,145,146,159,160]. - High-quality protein sources that are not lean include fatty fish, unsalted nuts, nutritional yeast, seeds (e.g., chia seeds, flax, and hemp), and eggs [161]. Avoid processed and red meats if possible [162]. - It should be noted that strength training and avoiding sedentary activity is more important for the preservation of lean mass than protein intake [163].

### 9.1. The Role of Diet Quality in the Treatment of Obesity

Beyond energy balance, dietary quality plays a pivotal role in determining long-term health outcomes. Diets emphasizing whole, minimally processed foods—such as vegetables, fruits, whole grains, legumes, nuts, seeds, and lean protein sources—have been consistently associated with a reduced risk of obesity, type 2 diabetes, hypertension, and cardiovascular disease [164,165,166,167]. In contrast, the frequent consumption of ultra-processed foods is linked to metabolic dysregulation, inflammation, and adverse body composition, even independent of caloric intake [168,169,170].

In recent decades, dietary patterns have shifted toward an increased consumption of processed and ultra-processed foods. These products, often rich in added sugars, refined grains, unhealthy fats, artificial sweeteners, and food additives, are engineered for hyper-palatability and typically lack fiber and essential nutrients [171,172,173]. Ultra-processed foods now account for a substantial proportion of daily energy intake in many high-income countries and have been strongly associated with weight gain, insulin resistance, cardiovascular disease, and all-cause mortality [168,169,174]. Diets high in added sugars, artificial sweeteners, and sugar alcohols, often found in ultra-processed products, have been associated with gastrointestinal discomfort—including gas, bloating, and diarrhea—and in some studies, increased hunger and a potential link to obesity [148,169,171,173,175]. One notable exception is refined grain. Refined grain products, which are consumed more often in higher carbohydrate, low-fat diets, have received much negative press in the media in recent years. However, while whole grains are protective [176], with the exception of smaller studies in certain subgroups [177], refined grain products are not linked with increased risk of coronary heart disease, heart failure, all-cause mortality [176], or type 2 diabetes [177] in prospective cohort studies. However, refined grain products are often higher in calorie density and so patients should still be steered to whole grains.

Improving dietary quality requires a twofold approach: minimizing harmful components while increasing the intake of nutrient-dense, high-fiber foods, found most commonly in plant-based foods, plays a key role in supporting gut microbiota diversity, enhancing satiety, and improving glycemic control [178]. Additionally, patients should be counseled to choose carbohydrates from cooked whole intact grains, (grains containing the bran, endosperm, and germ in their whole form rather than ground into flour) and cooked beans as well as starchy vegetables to reduce the calorie density of their diet. These have a low caloric density (0.6–1.5 kcal/g), have lower glycemic loads, and promote satiety better than processed carbohydrate foods. These foods can stimulate natural GLP1 production, albeit at much lower levels than pharmaceuticals and bariatric surgery [154,155]. Given the cardiometabolic benefits of eating foods that are high in fiber, anti-oxidants, and phytonutrients, such as fruits, vegetables, beans, and whole grains, regardless of the dietary pattern a patient follows, the recommendation should be to include more plants [179].

Protein quality and source also influence health outcomes [180]. While protein intake tends to remain relatively stable across different diets [140,157], evidence suggests that high-quality protein—particularly from whole foods or select supplementation—can support body composition, weight maintenance, and satiety in individuals with overweight or obesity [137,159,160,181]. Those consuming animal-based protein should be encouraged to eat lean protein options and steered toward lower fat sources of animal protein such as fish/seafood and poultry [71]. Processed and red meats have also been linked to increased health risks and increased weight [162].

### 9.2. The Role of Dietary Pattern

While caloric deficit is a common theme of dietary-induced weight loss interventions, there are a variety of sustainable dietary patterns for patients to choose from as no particular macronutrient breakdown has been shown to be generally superior for weight loss, with adherence to behavioral therapy sessions being a greater predictor for better outcomes than macronutrient breakdown [16,18,182]. Below, we explain considerations for different types of dietary patterns in recommendations for patients with obesity. Table 7 provides a summary of various dietary patterns with pros and cautions as one of is considering a recommendation for various patients.

### 9.3. Volumetrics Approach

The primary goal of a volumetrics diet is to induce a sensation of fullness while maintaining caloric restriction through the consumption of high volumes of nutrient-rich and low-calorie foods (i.e., reduced energy density in calories per gram or calories per pound) [156]. A summary of the calorie density of various foods can be found in Table 6. Volumetrics diets have shown to be helpful for weight loss and can provide an avenue for individuals to feel satisfied even when restricting calories [72]. Volumetric diets might be helpful for patients who like eating large portions of food and for patients who prefer not to track calories. Volumetrics principles can be applied to many dietary patterns. Handouts on energy density can be valuable for teaching patients principles of healthy nutrition. Volumetrics principles can be applied to any dietary pattern and can be a useful approach to assist patients. The Full Plate Living program is one resource that provides education on volumetrics and calorie density-based principles [206].

### 9.4. Low-Fat and Plant-Based Diet

Low-fat diets prescribe less than 30% of calories from fat. The DASH diet is likely one of the most well-known high carbohydrate, low-fat diets modeled after the “healthy vegetarian diet” in the 1980s and 90s [207]. The DASH diet is associated with a decreased incidence of cardiovascular disease, coronary heart disease, stroke, and diabetes in prospective studies and decreased blood pressure, LDL, hemoglobin A1C, fasting insulin, and body weight [164]. Very-low-fat vegan diets with less than 10% fat have been shown to improve body weight, hepatocellular lipid levels, and insulin sensitivity as well as induce diabetes remission in randomized trials [191,192]. However, reducing saturated fat without considering the replacement macronutrient can lead to an increased intake of added sugars and ultra-processed foods, resulting in adverse metabolic effects, including an increased risk of coronary heart disease and type 2 diabetes [208,209,210].

Plant-based diets are those that emphasize foods primarily from whole or minimally processed plant foods including vegetables, fruits, whole grains, nuts, seeds, beans, and other legumes. Plant-based diets, as a broad term, might represent a spectrum of diets including vegetarian, pescatarian, flexitarian, DASH, MIND, Mediterranean [183,184], vegan, or whole food plant-based (WFPB) diets [144,185,211]. The common thread in these diets is the emphasis on minimally processed whole plant foods and a limitation of animal-based and refined/ultra-processed foods. These dietary patterns are naturally high in quality and low in calorie density as they are founded on fruits and vegetables, legumes, and whole grains, moderate or more limited amounts of nuts and seeds, and the limitation of added sugars and oils. Individuals consuming more plant-based diets have lower rates of overweight and obesity than those whose diets include or emphasize meat and refined foods [212]. Though some plant based diets emphasize healthy fats, plant-based patterns are generally lower in fat and cholesterol and promote both weight loss and long-term healthy weight maintenance since they follow volumetrics principles, outlined above, which help to combat hunger [71,185,186]. Plant-based diets have been shown to be effective for weight loss and maintenance [144,185,211].

Well-planned plant-based or low-fat diets for weight loss should include appropriate protein intake to minimize muscle loss. Patients on such a diet should be counseled to increase omega-3 fatty acids, vitamin B12, vitamin D, iron, zinc, iodine, and calcium [213].

### 9.5. Low-Carbohydrate Diet

Low-carbohydrate diets prescribe 60–130 g of carbohydrates per day (≤20–45% of daily energy intake), with many recommending less than 100 g per day [214]. Very-low-carbohydrate regimens recommend less than 60 g of carbohydrate per day [214]. Calorically restricted low-carbohydrate diets have been shown to be effective for weight loss [182,215]. One of the most popular options for a low-carb diet is the very-low-carbohydrate ketogenic diet, where carbohydrate intake is typically restricted to less than 50 g per day [214]. This diet has been associated with significant weight loss and improvements in several metabolic parameters [179,215,216,217]. Any diet restricting a particular macronutrient should be well-planned. Due to the emphasis on higher protein or higher fat foods, often from animal products, low-carbohydrate diets may include higher levels of commonly overconsumed and less healthful saturated fat, cholesterol, and sodium, which can increase mortality [187]. The restriction of overall carbohydrates in the diet may also lead to a lower intake of healthful vitamins and nutrients, as well as fiber, antioxidants, and phytonutrients that are found primarily in fruits, vegetables, whole grains, and legumes.

When counseling patients who would like to embark on very-low-carbohydrate diets, the work of Volek and Phinney, whose diets have been studied in clinical trials, can be helpful in guiding patients toward a well-planned ketogenic diet [190]. Many diets that are considered “paleolithic”, such as the proprietary Whole 30^®^, are often moderately low carbohydrate while also still recommending many nutrient-dense foods [218].

One concern with recommending a ketogenic diet or low-carbohydrate diet profile is that the effect of low-carbohydrate diets on mortality is unclear. A systematic review by Shan et al. using NHANES data suggested that when low-carbohydrate diets focus on the consumption of fat from healthier sources this seems to improve mortality [187]. Large cohort studies suggest higher mortality at lower carbohydrate levels, but this association disappears when plant-based protein is substituted for animal-based sources of protein [188,189]. Given this, patients on ketogenic diets can be counseled to include plant based sources of protein as well as low-carbohydrate sources of soluble and insoluble fiber.

The American Diabetes Association advises that individuals with diabetes on SGLT-2 inhibitors should avoid low-carbohydrate ketogenic diets due to the risk of euglycemic diabetic ketoacidosis [152]. Those on ketogenic diets should also be counseled on ensuring proper sodium and potassium balance as ketogenic diets can cause natriuresis, which can also trigger potassium loss. Those in nutritional ketosis should be counseled not to eat low sodium diets and if in true nutritional ketosis, they should be counseled to consume up to 1–2 g of additional sodium and adequate potassium through food sources [190].

In some cases, those on a ketogenic diet may experience an increase in LDL substantial enough to impact CVD risk. After confirming a continued elevation of LDL with a repeat lipid panel, other causes of hyperlipidemia such as diabetes mellitus, hypothyroidism, nephrotic syndrome, liver disease, and recent changes in medications that may worsen cholesterol levels (such as some beta-blockers, corticosteroids, amiodarone, cyclosporin, anabolic steroids, protease inhibitors, and some diuretics), a trial off of the ketogenic diet may be recommended. Other approaches to reducing LDL in ketogenic diets can include replacing animal fats with plant fats, and increasing low-carbohydrate sources of fiber [179]. The eco-Atkins study diet, which used a vegan Atkins-style diet [219], and the OMNIHEART study diet, which provided a moderately low-carb diet by substituting 10% of calories from carbohydrate with either protein or unsaturated fat [220], echo this approach and showed weight loss and an improvement of cardiometabolic markers.

### 9.6. Very-Low-Calorie Diets (VLCDs)

VLCDs have a number of definitions, and typically consist of a caloric restriction of 800–1000 calories per day or less. These diets are often used to induce acute weight loss and are not meant to be maintained for a lifetime. VLCDs are typically achieved through the use of meal replacement bars, shakes, soups, and drink mixes that include full daily values of vitamins and minerals. They are often low-fat and high in protein, but some VLCD protocols follow a ketogenic approach [197]. VLCDs are very effective at achieving weight loss and significant improvement in cardiometabolic parameters in short periods [221,222], including diabetes remission, which can be helpful for patients needing fast results [199,223]. The DiRECT (Diabetes Remission Clinical Trial), used very-low-calorie diets with meal replacements, in which almost half of the study participants were able to achieve diabetes remission [198]. VLCDs should be prescribed only to carefully selected individuals by trained practitioners in medical settings with close monitoring due to the risk of side effects such as nutrient deficiencies, electrolyte abnormalities, arrhythmias, cholecystitis, and severe fatigue [53,179]. Long-term weight maintenance strategies and counseling should be integrated to maintain weight loss to prevent weight regain. One disadvantage of very-low-calorie diet programs is that patients should be ready to pay for such a program and then be able to continue to pay for some kind of a maintenance plan, and the cost can be prohibitive for some patients.

### 9.7. Time-Restricted Eating

Time-restricted eating (TRE), colloquially used interchangeably with the term intermittent fasting, is a practice of limiting food to a specified time window per day, such as 8–10 h. Individuals may stop eating, for example at 8 pm and start breakfast again the next day at 8 am. Some TRE regimens recommend as little as a six hour window of eating, such as the one-meal-a-day (OMAD) approach. A randomized trial of 139 patients with obesity did not show benefit over continuous calorie restriction [224], and this has fueled the hypothesis that TRE works primarily through calorie restriction. However, a counter-argument has suggested that fasting appears to turn on cellular pathways that may mediate the benefit of calorie restriction [200], and calorie restriction can also inadvertently reduce the time window during which people eat [202]. The safety and efficacy of smaller fasting windows of less than 8 h in free-living individuals is not clear at this time, though a small human study did show that a eucaloric diet of one meal a day did not reduce lean mass or aerobic capacity but did reduce fat mass [225]. While intermittent fasting can be safe and effective for patients with type 2 diabetes, they should have medications adjusted if they are on insulin secretagogues or insulin to reduce the risk of hypoglycemia, and blood sugar should be very closely monitored [152,204,205].

Patients at risk of acute kidney injury or those on diuretics should be counseled on adequate hydration during the fasting window and, based on medical conditions influenced by sodium and potassium balance, be counseled to incorporate electrolyte-rich drinks. As with ketogenic diets, patients with diabetes on SGLT2 inhibitors are at a higher risk of diabetic ketoacidosis and fasting may not be advisable. One useful aspect of time-restricted eating is that much like fasting, it can be layered on top of any dietary pattern for added benefit. A useful resource for those practicing time-restricted eating is the Salk Institute’s “MyCircadianClock” app by the Satchin Panda lab.

### 9.8. Fasting

The approaches to fasting with the most evidence are alternate day fasting or the 5:2 diet (2 days of fasting in a week), which has been found to be relatively safe [201,226]. Patients on a 5:2 diet are often on a low-carbohydrate, high-protein, and high-fat diet with lower fiber and at risk for some micronutrient deficiencies [226]; therefore, it can be useful to encourage eating more fruit, vegetables, whole grains, and legumes in a patient-centered discussion. It has been shown in randomized trials to be helpful in reducing indicators of liver fat content [227], multiple cardiometabolic markers, body composition, and insulin resistance including in type 2 diabetes. [203]. Fasting poses an increased risk of diabetic ketoacidosis (DKA) for patients with type 1 diabetes, and euglycemic DKA for those with type 2 diabetes, particularly those on SGLT-2 inhibitors, and poses a higher risk of hypoglycemia. [152,228]. The American Diabetes Association recommends against the use of fasting in patients on SGLT-2 inhibitors [152]. Those on anti-hyperglycemic medications, particularly insulin secretagogues, diuretics, anti-hypertensives, and other medications that affect electrolyte balance may need medication adjustments [203]. Fasting and shorter eating windows may predispose to more lean mass losses. Patients should be screened for signs of sarcopenia and risk for nutritional deficiency before embarking on any kind of fasting [203]. Prolonged fasting with a duration of more than 3 days has metabolic benefits, though the majority of weight loss may be lean mass [229]. The safety is still to be determined [203,229].

### 9.9. Fasting-Mimicking Diets

These diets aim to mimic the metabolic effects of fasting while allowing some food intake. Studies have shown that short cycles of very-low-calorie intake, such as those used in FMDs, can lead to decreases in fat mass while preserving lean mass, improved physical performance, and glucoregulation [230,231]. However, the long-term benefits and safety of FMDs require further research. They often use periodic, proprietary 5-day protocols using meal replacements which can be convenient, but potentially prohibitively expensive for patients [230].

## 10. The Role of Physical Activity in Obesity Treatment

### 10.1. Role of Aerobic Exercise in Obesity Treatment

While diet is a crucial factor in managing obesity, physical activity plays an indispensable role [53,232]. Although meeting public health recommendations for physical activity is only associated with modest weight loss [233,234], it markedly improves cardiometabolic health [235,236]. Studies have shown that aerobic exercise can lead to an additional weight loss of 2–3 kg on average compared to no exercise [237,238].

Cardiovascular exercise/aerobic training is effective in reducing abdominal visceral fat and is crucial for improving cardiometabolic health [237]. For meaningful weight and total adiposity loss, increasing activity to a minimum of 225 (and up to 420) minutes per week of aerobic activity of at least moderate intensity is recommended, and for optimal results should be combined with dietary change [234,239]. However, as discussed in the section on injury, patients should achieve this goal gradually.

Acute exercise can cause reductions in overall energy intake that can cause weight gain, so prescriptions for physical activity should be provided with a concurrent recommendation to continue to keep overall activity constant with attention to step counts and increasing non-exercise activity thermogenesis [240]. However, given the significant benefits of exercise, this should not be a reason to avoid exercise and physical activity for those with obesity. Aerobic exercise improves cardiorespiratory fitness/VO_2_ max, which has been shown to be one of the strongest predictors of all-cause mortality [238,241]. This improvement in cardiorespiratory fitness is notably independent of weight loss and contributes significantly to overall health benefits. Additionally, moderate to vigorous physical activity makes many contributions to overall health such as improvements in mental health and overall physical function, and reduces risk for myriad diseases [234,235]. Many patients with obesity are seeking to avoid physical disability as they age, and moderate to vigorous physical activity including both aerobic and strength training, as well as an active lifestyle, is an important way to ensure this occurs [242].

Aerobic exercise training can also be helpful for those going through bariatric surgery. Exercise following surgery may synergistically enhance weight maintenance, glycemic management, and insulin sensitivity [243], lower the risk of cardiovascular disease, enhance endothelial function, and improve cardiac autonomic regulation [244]. Overall, physical activity can induce and maintain improved health-related quality of life for up to 2 years after RYGB and should be encouraged. [115].

An approach to increasing moderate to vigorous physical activity gradually is the use of physical activity snacks—short bouts of moderate to vigorous physical activity lasting 2–5 min. There are benefits for BMI [245], the preservation of skeletal muscle mass, muscle strength, cardiorespiratory fitness [246], and insulin resistance [247].

### 10.2. Role of Strength Training in Preventing Muscle Loss

Sarcopenia is defined as an age- or disease-related loss of healthy muscle mass and function [248,249]. Effective weight loss strategies causing individuals to undergo significant weight loss often also cause a significant loss of fat-free mass [250,251]. Individuals with obesity, particularly at-risk populations such as post-menopausal women, elderly, those with significant disability, those with metabolic disease including diabetes, and athletes, are often contending with a loss of fat-free mass when undergoing weight loss interventions of anywhere from 5–35% [163,250]. In addition to this, their baseline muscle quality is already poorer compared to individuals without obesity [252]. Sarcopenia is associated with physical disability, poor quality of life, and increased mortality risk [253,254]. Sarcopenic obesity poses increased cardiovascular risk and accelerated functional decline [140,255,256].

Estimates from multiple clinical trials suggest that fat-free mass loss from GLP-1RA medications can be anywhere from 25 to 40% of the total weight loss [251]. The clinical significance of this fat-free mass loss is yet unclear, given that function is preserved and muscle quality often improves weight loss, including medically and surgically induced weight loss [250]. However, patients with obesity at a higher risk of sarcopenia should be counseled on mitigating factors due to the risks of sarcopenia discussed above. While medications to mitigate muscle loss are being investigated, increased protein intake and endurance and resistance training can have sarcopenia-mitigating effects [136,158,252,257].

While there are many protocols, the most studied approaches incorporate full-body workouts using predominantly compound movements for at least 10 weeks [258]. Studies have suggested an important factor for optimizing muscle protein synthesis and hypertrophy is achieving near contractile failure, defined as maximum muscle fatigue and motor unit activation [257]. The optimal frequency of training is still relatively unknown, but many studies conclude a 2–3 day per week frequency as an effective floor for achieving muscle loss prevention benefits [136]. Therefore, a practical approach to strength training to prevent sarcopenia can include a periodized, progressive resistance exercise protocol that emphasizes functional compound movements performed 2–3 times per week. Optimal protein intake is discussed elsewhere in this article.

Exercise can also prevent muscle loss in bariatric surgery. Patients who undergo bariatric surgery are particularly susceptible to skeletal muscle loss or sarcopenia [253,254]. Biweekly physical activity training sessions for 6 months after Roux-en-Y gastric bypass (RYGB) have been shown to improve cardiometabolic risk factors and muscle strength. However, patients may not maintain these benefits in follow-up without continued exercise [115,259,260]. According to a recent systematic review and meta-analysis, exercise training leads to a large increase in muscle strength after bariatric surgery compared with a non-exercise control group [261].

### 10.3. Preventing Injury in Patients with Obesity Who Are Prescribed Exercise

Ensuring patients can exercise safely and effectively is key to managing obesity. Doing this requires tailoring the exercise prescription to a patient’s abilities, preferences, available resources, and comfort level to ensure sustainability. Individuals with obesity or overweight may be at higher risk of injury when starting an exercise plan [262,263]. Understanding and setting reasonable expectations remains incredibly important to align with healthcare providers and patients’ needs and goals to mitigate risks, including injury.

Pre-emptive strengthening exercises targeting the foot, ankle, hip abductors, quadriceps, and trunk/core can support joints and reduce injury risk in some contexts [264,265,266]. Starting with low-impact activities such as walking, swimming, or cycling can help minimize joint stress and allow for a gradual increase in intensity and duration. These activities have been found to be effective interventions in those with established pain and osteoarthritis [267,268].

## 11. Weight Maintenance and Preventing Metabolic Adaptation

Dietary, physical activity, and behavioral recommendations for weight maintenance are less clear and a subject for further research. People with obesity may experience extremely high rates of weight regain after an initial weight loss [269]. At least some of this has been attributed to metabolic adaptation, also termed adaptive thermogenesis in the literature. While medications or surgery often play a role in mitigating weight regain, lifestyle measures can be implemented to mitigate weight regain.

Protein seems to have a beneficial effect on weight maintenance. Higher protein diets (1.2–1.6 g/kg of adjusted bodyweight versus standard 0.8 g/kg) may prevent this type of metabolic adaptation [141,270], but this may be difficult for some patients to adhere to due to the challenge of maintaining a lower calorie intake while eating high-protein foods, gastrointestinal side effects, or taste preferences [196]. Other dietary methods of addressing metabolic adaptation include ensuring high fiber intake and intermittent energy restriction. High fiber intake may work to mitigate metabolic adaptation through the upregulation of orexigenic gut hormones, reduction of energy density, and slowed gastric emptying. Intermittent energy restriction has been shown in small studies to preserve fat-free mass, and may work through the reduction of muscle protein breakdown due to acute carbohydrate refeeding and through increased leptin levels during increased carbohydrate intake [271,272,273].

Ongoing interactions and group settings improve weight maintenance after weight loss with lifestyle intervention. The 2013 obesity treatment guidelines from The Obesity Society, the American College of Cardiology and the American Heart Association recommend long-term comprehensive weight maintenance programs [236]. Behavioral counseling may include, as with during the weight loss phase, counseling on continuing behaviors associated with long-term weight loss such as self-monitoring, self-weighing, reduced calorie intake, more frequent meals and snacks throughout the day, increased physical activity, consistently eating breakfast, reducing meals outside the home and fast food meals, reduced screen time, and the use of portion-controlled meals and meal substitutes [270].

Some behavioral approaches to weight loss that have been shown to be helpful for preventing weight regain include strengthening satisfaction with outcomes, relapse prevention training, cognitive restructuring, developing cognitive flexibility, appealing to deeper motivations, and managing expectations [270]. When patients reach plateaus or regain weight, a patient-centered conversation may be had with patients on escalating treatment vs. continuing on the current path, taking into account the stage of obesity, comorbidities, quality of life, and patient goals [270].

Physical activity is effective for weight management not only in maintaining weight loss but also in preventing the worsening of obesity as individuals age. Moderate to vigorous physical activity is associated with a slower decline in BMI over time in middle-aged and older adults, suggesting that maintaining and/or increasing physical activity levels can help manage body weight effectively as one ages [274]. Additionally, exercise improves fat-free mass and drives metabolic integration between muscle, adipose, and liver metabolism, which helps to maintain metabolic homeostasis and protect against age-related metabolic diseases [275,276].

As discussed in the previous section, it is common for patients to lose both adipose tissue and lean tissue during weight loss interventions. Lean tissue is healthier and uses more energy than adipose tissue with one pound of muscle burning 6 kcals per day and one pound of fat burning 2 kcals per day [277]; preserving lean tissue during active weight loss periods can help prevent metabolic adaptation, where metabolism slows down and resting energy expenditure lowers, making it harder for patients to maintain weight loss long term. More gradual weight loss has been shown to reduce fat mass and preserve lean mass [278]. If additional calories are consumed, a progressive resistance training program, as discussed above in the section on preventing sarcopenia, can encourage the additional energy to be put towards increasing muscle mass, as opposed to being stored primarily as fat mass.

Evidence suggests that higher levels of physical activity are associated with better weight maintenance outcomes. Proving a causative relationship between exercise and weight maintenance remains challenging, however, due to the complexity of multiple factors involved in weight loss. [279]. Greater than 150 min (and preferably up to 250 or 300 min) per week of aerobic activity of at least moderate intensity is often required to prevent weight regain after weight loss [235,239]. Retrospective analyses indicate that relatively high volumes of exercise are needed for weight maintenance [237]. This is further supported by the prominence of physical activities in the role of weight maintenance for participants in weight control registries in the US and other countries [280]. Despite the limitations in the available studies, the consensus is that sustained physical activity is a critical component of long-term weight management strategies.

Increasing physical activity by encouraging an active lifestyle by increasing non-exercise activity thermogenesis (NEAT) proactively through step counters or increasing household tasks can be especially helpful to individuals who are sedentary due to a sedentary occupation, disability, or other reason. NEAT can increase energy flux while not causing individuals to reduce their net energy expenditure as moderate to vigorous physical activity is known to [32,240,281]. However, while evidence from the national weight loss registry does suggest participants kept weight off through increased walking [280], there is little research pointing to interventions specifically increasing NEAT as a way to maintain weight, with this being a research gap overall [281,282].

High-intensity interval training over the course of 22 min a day can significantly increase energy expenditure compared to continuous exercise. Regular endurance exercise may improve metabolic flexibility through mechanisms mediated by sympathetic nervous system support of increased resting energy expenditure [281]. Regular exercise may also cause the browning of white adipose tissue and increase the activation of brown adipose tissue, which, regardless of weight loss, has been associated with improved metabolic outcomes [283].

Ongoing communication regarding a patient’s experience is also important for sustaining an exercise plan in the long term. As discussed under the section on stigma, a focus on intrinsic motivators such as health rather than extrinsic motivations like weight loss and appearance can be helpful [65]. This can help prevent frustration and the cessation of exercise due to feeling that it is “ineffective” in helping them lose weight. Given that less than one in four individuals meet the physical activity guidelines for both aerobic activity and resistance training [284], sustaining levels above this from individuals who are obese or overweight can be challenging. In studies focused on dropout rates in exercise programs in different settings, unsurprisingly, adherence remains low, though appropriate supervision and the consideration of mHealth technology have promise [285,286,287,288,289]. Given these challenges, ongoing engagement with healthcare providers such as primary care providers, health coaches, exercise physiologists, and physical therapists remains critical to both contextualize patients’ frustrations but also provide ongoing motivation.

## 12. Clinical Models to Support Lifestyle Medicine Treatment Access for Patients with Obesity

Clinicians in the field of lifestyle medicine leverage a variety of fee-for-service reimbursed clinical delivery models to address chronic conditions including obesity.

### 12.1. Shared Medical Appointments

Shared medical appointments (SMAs) or group medical visits are individual healthcare visits delivered in a group setting. SMAs are often offered to groups of patients to address a specific chronic condition, like obesity [290,291], but cohorts may also consist of patients with a variety of conditions [292].

SMAs can allow for a more efficient treatment of chronic conditions and delivery of patient education; they can improve access to care, can increase social and clinical care support, and are linked to improved patient outcomes [292,293] and patient experience [292] as well as provider satisfaction [293].

Typically, SMAs are delivered as individual medical visits within a group setting and may leverage planned curriculum/education around chronic conditions. Complementary SMA curricula exist that might benefit patients with obesity, including the Ardmore Institute of Health Full Plate Living Program focused on increasing fiber-rich foods (https://www.fullplateliving.org/ accessed on 5 July 2025) and the UC San Diego SLIM Curriculum focused on weight management (https://slim.ucsd.edu/ accessed on 5 July 2025).

Providers (MD/DO/NP/PAs) often co-lead SMAs with a behaviorist and a medical assistant or nurse to support administrative needs and patient check-ins. Cohorts of 8–12 patients are common, and the frequency of visits is often 4–12 and even up to 16, with visit lengths varying from 60 to 120 min. Since there is no standard SMA model, these groups are often tailored to the needs of the patient populations they serve, settings where they are delivered, and resources available [291].

SMAs are most often billed as E&M visits (99213-99214) based on medical decision making. Though clear weight loss outcomes are still to be determined, SMAs and group interventions have proven to be a feasible delivery model for the treatment of obesity, including the provision of intensive behavioral therapy [290,291,294,295].

### 12.2. Medical Nutrition Therapy

Medical nutrition therapy is a nutrition-based treatment provided by a registered dietitian nutritionist (RDN). It includes an assessment and nutrition diagnosis with an intervention followed by appropriate monitoring and evaluation. At the federal level, CMS reimburses MNT delivered by RDNs for type 2 diabetes and chronic kidney disease [296]. Various states and health plans offer MNT for other chronic conditions as well. MNT can be provided individually or within a group using CPT codes 97802-97804. One approach to making MNT more accessible may be to offer group MNT at a lower “self-pay” rate. MNT should be administered by trained dietitians as part of an interdisciplinary team and should aim to achieve positive health outcomes along with weight changes [297].

### 12.3. Collaborative Care Management (CoCM)

CoCM is an integrated care model developed to treat common mental health conditions within primary care settings. It uses a team-based approach, including a primary care provider, a behavioral health care manager, and a psychiatric consultant. CCM services can also help reduce geographic and racial or ethnic health care disparities. CoCM can support primary care teams in helping patients with mental health conditions that may be impacting their ability to lose or maintain weight loss.

### 12.4. Remote Patient Monitoring

Remote patient monitoring (RPM), also known as remote physiologic monitoring, uses digital technologies to monitor and capture medical and other health data from patients. This information is then electronically transmitted to healthcare providers for assessment and, when necessary, to provide recommendations and instructions [298]. RPM programs employ various types of devices, including weight scales, pulse oximeters, blood glucose meters, blood pressure monitors, heart monitors, and wearables (such as smartwatches and continuous blood glucose monitors) [298]. Devices used must meet the definition of a medical device by the FDA [298]. RPM has been found to be helpful for a variety of outcomes [299]. RPM may help patients stay accountable as they measure progress toward and the achievement of weight loss goals, but more evidence is needed [300].

### 12.5. Chronic Care Management

Chronic care management (CCM) is a care management service designed for patients that have multiple (two or more) chronic conditions that are expected to last at least 12 months [301]. These services are not typically face-to-face and allow eligible practitioners to bill at least 20 min or more of care coordination services per month [301]. CCM includes a coordinated care plan that includes health checks, regular screenings, and long-term management. CCM can be billed on a monthly basis by a designated health professional in a practice [301]. CCM can be a great opportunity to check in on care plans and maintain accountability for patients with obesity and other chronic conditions [302], though some up-front investment and time is required on behalf of healthcare providers [303].

### 12.6. Intensive Behavioral Therapy

Intensive behavioral therapy (IBT) for obesity allows Medicare participants who meet the BMI criteria of ≥30 kg/m^2^ up to 22, 15 min IBT counseling sessions per 12-month period. These visits should be delivered at specific intervals and must be delivered by or “incident to” a physician or practitioner who is present at the time of the visit. IBT can be delivered individually or in a group of 2–10 patients [49]. An IBT protocol developed for primary care has demonstrated effective weight loss and may be a great guide to use with eligible patients [51]. IBT delivered by RDNs incident to physicians has been shown to improve A1C and medication use in a primary care setting [304]. The implementation of these codes has been challenging due to strict criteria and low reimbursement levels [305].

## 13. Role of the Multidisciplinary Team in Lifestyle Medicine Treatment of Obesity

The multidisciplinary team (MDT) essential for obesity management, especially when addressing the six lifestyle medicine pillars, with or without pharmacotherapy or surgery, would ideally consist of a nutrition professional, exercise physiologist, occupational therapist, physical therapist, pharmacist, and health coach.

**Figure 1:**
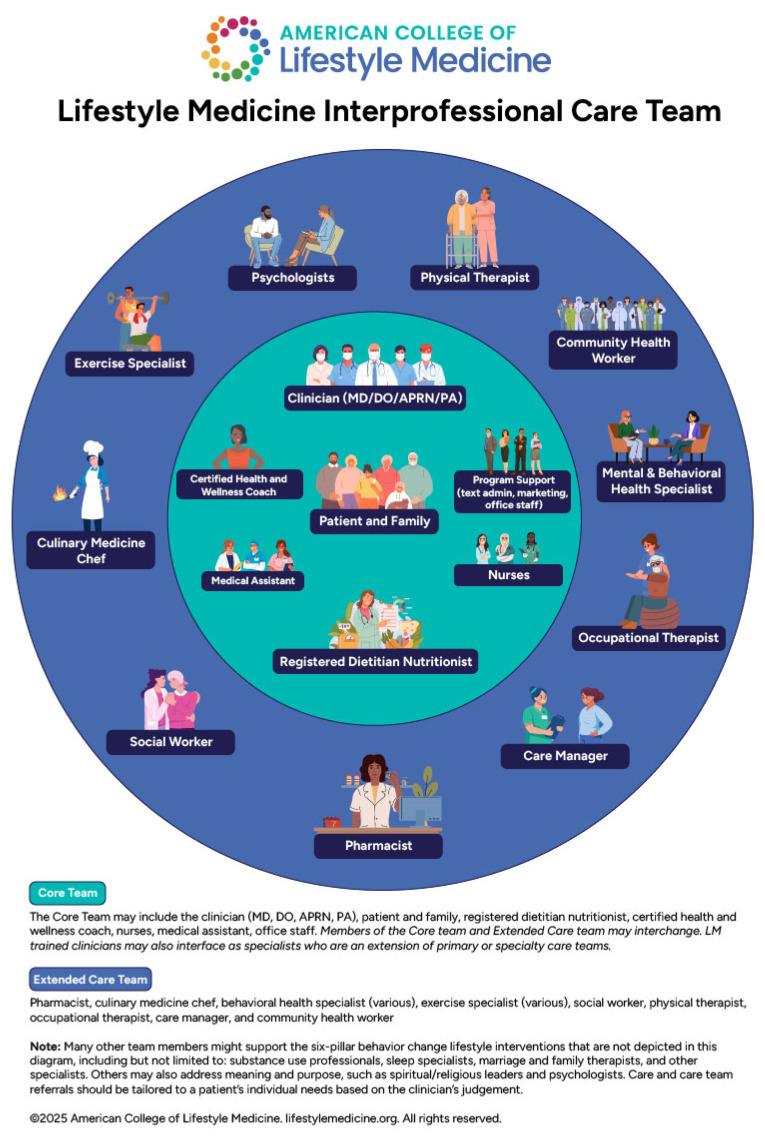
Lifestyle medicine interprofessional care team.

Most providers feel unprepared to manage obesity without an MDT approach because they either do not have specialized training in these disciplines and/or the time necessary to counsel and guide the patient appropriately. A systematic review by Tapsell and Neale found that a multidisciplinary intervention with physician oversight produced greater and more clinically significant weight loss. Additionally, these weight loss programs have shown improvement in areas other than weight, such as eating behaviors, lipid profiles, aerobic capacity, and overall quality of life [306]. Below, we have described the role of some of the potential interdisciplinary team members in a healthcare team that integrates lifestyle medicine. A more comprehensive but not exhaustive representation of the make-up of a lifestyle medicine team is provided in Figure 1.

### 13.1. Exercise Physiologist/Certified Personal Trainer

Exercise professionals offer specialized expertise in exercise prescription and lifestyle modification. Exercise physiologists develop tailored exercise programs that consider a patient’s fitness level and personal goals [307,308]. This specialty has become all the more relevant to combat the loss of lean muscle mass reported with the GLP-1 RA medications, since combining GLP-1 RA therapy with a structured exercise regimen enhances fat loss while preserving lean muscle mass [252].

### 13.2. Psychologist

A psychologist is necessary on any bariatric surgical team to ensure that a patient does not have eating disorders and is suitable for surgery [114]. Psychologists can help manage mental health sequelae due to weight bias, disordered eating, or other underlying psychological conditions [33].

### 13.3. Health Coach

The health coach typically utilizes behavioral coaching techniques, motivational interviewing, goal setting, and continuous monitoring to guide patients toward lasting lifestyle changes [309]. Studies have shown that health coaching for obesity is mixed, but there have been some promising results [310,311,312,313,314] and future literature should focus on facilitators of improved outcomes within specific interventions. It should be noted that the Diabetes Prevention Program is provided by a lifestyle coach [315]. Health coaching in lifestyle medicine factors such as diet and physical activity has been shown to increase adherence to behavioral changes and assist with obesity if delivered as part of an intensive, multicomponent program [19,316]. A barrier to access is that health coaching is not covered by insurance. Group coaching using self-pay models may be considered as an approach to improving accessibility.

### 13.4. Occupational Therapist (OT)

Obesity can impact a person’s ability to perform self-care tasks, such as dressing, bathing, and cooking. OTs help patients develop healthier habits, modify daily routines, and enhance their ability to perform activities of daily living (ADLs) and work-related tasks. Many people with obesity face limitations in mobility, flexibility, or endurance, which can make it difficult for them to engage in exercise or even perform daily tasks. OTs help individuals modify their environments, by recommending assistive devices or suggesting modifications in home, work, or community settings to increase participation in physical activity. Occupational therapy has been studied for weight loss in small trials with significant outcomes in the short, intermediate, and long term [317]. Some innovative programs have notably used OTs to provide lifestyle therapy.

### 13.5. Physical Therapists (PT)

Physical therapists provide comprehensive evaluations and design individualized exercise programs aimed at enhancing mobility, increasing physical activity levels, and promoting weight loss that are tailored to accommodate each patient’s unique needs and comorbidities, including those with complex chronic conditions [232]. They provide instruction on proper exercise techniques, posture, and body mechanics, empowering patients to incorporate physical activity into their daily routines confidently. This education fosters long-term adherence to healthy lifestyle changes. Obesity often leads to musculoskeletal injury [318] and functional impairment [319]. Physical therapists employ various interventions, such as manual therapy and therapeutic exercises, to alleviate pain and improve joint function. [320]. By addressing these issues, they enable patients to adhere to exercise regimens essential for weight management.

### 13.6. Pharmacists

Pharmacists can assess patients’ current medications to identify those that may contribute to weight gain and suggest alternatives. Pharmacists have a unique advantage of interacting with patients more often than other providers and are also able to titrate doses of anti-hyperglycemics and anti-hypertensives more frequently as the patient’s medical condition changes, sometimes necessitating rapid de-escalation during weight loss [321]. Some models of care have trained pharmacists on lifestyle modifications counseling, including diet and physical activity, tailored to individual needs [107]. They educate patients on the proper use of weight loss medications, potential side effects, and the importance of adherence to prescribed therapies. This education empowers patients to make informed decisions about their health [322].

In summary, in the age of highly effective obesity medications, an MDT approach has become even more important. Dietary and exercise counseling, in particular, is key to avoiding some of the adverse outcomes of muscle loss, malnutrition, and other metabolic changes that can occur with these medications. Integrating GLP-1 RAs and bariatric surgery with a multidisciplinary lifestyle medicine approach has demonstrated significant improvements in weight management, glycemic control, and overall metabolic health. [152]. This combined strategy leverages the benefits of medication and surgery alongside personalized lifestyle interventions to achieve superior health outcomes [323,324].

## 14. Summary and Conclusions

Even in an era of highly effective obesity treatments such as GLP-1 RAs and metabolic surgery, lifestyle medicine continues to be foundational to obesity treatment. Obesity care should be driven by stage, comorbidity, and patient preference and values that include lifestyle medicine treatment [15,26,115,152]. A patient’s decisions about their body and their weight should be respected [54,55,56]. Obesity continues to be a major public health problem and clinicians must be ready to address the health needs of people with obesity. Comprehensive obesity care includes understanding the role of lifestyle prescription in the treatment of obesity during weight loss and weight maintenance [12]. When therapy is escalated to pharmacotherapy or surgery, lifestyle approaches can help patients minimize side effects [325]. There are multiple care models to provide access to lifestyle medicine for patients with obesity and clinicians and health systems should strive to make these available for patients. When clinicians do not have the resources to provide comprehensive obesity care, they may refer to multidisciplinary team members who can close these gaps for patients [306].

## Figures and Tables

**Table 2:** Comprehensive Lifestyle Medicine Assessment.

Assessment Tools	- **Lifestyle Medicine Health Behavior Scale** [44]**.** - **Lifestyle Medicine Inventory** [45]**.** - **Lifestyle Medicine Vital Signs Questionnaire** [46]**.** - **American College of Lifestyle Medicine Short- and Long-form Questionnaires.**
Sleep	- Screen for **obstructive sleep apnea (OSA)**; refer for sleep study if undiagnosed. (Weight loss (≥10%) can significantly improve OSA (e.g., ~50% drop in AHI).) - Assess other sleep issues (e.g., insomnia or shift work); poor sleep quality contributes to weight gain. - Teach **sleep hygiene**. - Refer to **CBTi** (cognitive behavioral therapy for insomnia) when possible. o Alternative resources: VA CBTi Coach App and *Improve Your Sleep* self-guided workbook.
Substance Use	- **Alcohol**: Screen for alcohol use disorder; consumption can contribute to weight gain. Can worsen pre-existing MASH/MASLD. - **Tobacco**: Screen and support cessation as it can worsen pre-existing medical problems. - Quitting may lead to weight gain; monitor closely. - A history of substance use disorders may modify plan for type of anti-obesity medication that is started for the patient. Remission from substance abuse is a risk factor for weight gain.
Stress and Mental Health	- Assess for conditions like anxiety and depression that may lead to off-plan eating. - Refer to mental health providers. - Provide tools for stress management (e.g., diaphragmatic breathing, guided imagery, progressive muscle relaxation, hobbies, and social or spiritual practices). - Collaborate with a **multidisciplinary team** for appropriate referrals and support.

**Table 4:** Anti-obesity medications—side effects and lifestyle support strategies [52,87].

Medication Class	Examples	Common Side Effects	Lifestyle Support Strategies
GLP-1 Receptor Agonists	Semaglutide, Liraglutide	Nausea, constipation, bloating, early satiety, muscle loss	Small, low-fat meals; slow titration; encourage fiber and hydration; resistance training and ≥60 g protein/day to reduce lean mass loss
Stimulants (Sympathomimetics)	Phentermine, Benzphetamine	Insomnia, tachycardia, anxiety, elevated BP	Avoid evening dosing; monitor vitals; gradual exercise progression; avoid caffeine [87]
Noradrenergic/Dopaminergic Agents	Bupropion	Headache, dry mouth, insomnia, elevated BP	Hydration; morning dosing; stress management; monitor mood and cardiovascular status
Combination: Bupropion/Naltrexone	Contrave^®^	Nausea, vomiting, headache, mood changes	Take with food; hydration; consider mental health screening and support
Phentermine/Topiramate (Anticonvulsant)	Qsymia^®^ (topiramate/phentermine)	Dizziness, cognitive effects, paresthesia, heat intolerance	Avoid solo high-risk activity; ensure hydration; caution with heat exposure; screen for cognitive changes; foods containing vitamin C or vitamin C supplementation may mitigate side effects of nephrolithiasis [108]
Orlistat (Lipase Inhibitor)	Xenical^®^, Alli^®^	GI urgency, oily stools, fat-soluble vitamin loss	Recommend low-fat diet (<30% fat); supplement A, D, E, and K; schedule physical activity post-GI relief
GIP/GLP-1 Dual Agonists	Tirzepatide	Similar to GLP-1 RA: GI symptoms, possible hypotension	Monitor hydration, encourage water-rich foods; titrate activity based on tolerance [87,104,105]

**Table 5:** Lifestyle medicine interventions in the setting of metabolic surgery.

Phase	Lifestyle Focus	Key Interventions
Preoperative	Nutritional optimization, behavior readiness, and comorbidity management	RD evaluation, supervised weight loss, CBT/psychological evaluation, micronutrient screening [98,99,100,101,102,103,104,105,106]
Immediate Postoperative	Recovery, hydration, nutrient absorption, and complication monitoring	Advance diet stages; monitor protein and fluid intake; gentle activity; supplement adherence
Long-term Maintenance	Preventing weight regain, supporting metabolic health, and bone/muscle preservation	≥60 g protein/day; progressive exercise (cardio + resistance); monitor for B12, iron, and D; regular follow-up [109,110,111,112,113,114,115,116,117,118,119]

**Table 7:** Summary of common dietary patterns in obesity management.

Diet Type	Key Features	Pros	Cautions
General principles	- In general, the patient should be encouraged to follow a dietary pattern that they can adhere to, while encouraging improvement in diet quality, including foods high in fiber and water while ensuring adequate protein [12,16,52,179].		
Plant-based	- Plant-based diets, as a broad term, might represent a spectrum of diets including vegetarian, pescatarian, flexitarian, DASH, MIND, Mediterranean [183,184], vegan, or whole food plant-based (WFPB) diets. - Emphasizes whole plant foods: vegetables, fruits, legumes, and whole grains; minimizes animal products and ultra-processed foods.	Improves cardiometabolic health; promotes satiety; and reduces inflammation and insulin resistance. With emphasis on low-calorie-density foods, can provide weight loss while minimizing hunger and restrictive approaches [71,185,186].	Risk of B12 and iron deficiency without supplementation [179]. Ensure adequate protein.
Volumetrics	- Focus on high-volume, low-calorie-density foods (soups, salads, fruits, vegetables, cooked starchy vegetables, beans, and grains). - Exercise portion control with higher-calorie-density foods. See Table 6 for more information.	Increases fullness without strict calorie tracking; adaptable to many diets. Reduces hunger during calorie restriction [72].	May require meal prep and education.
Low-carbohydrate	- <130 g/day; ketogenic typically <50 g/day; higher fat and/or protein.	- May help with glycemic control when patients achieve weight loss [179]. - Satiety from higher protein and palatability from fat may make this easier to follow for some.	- Potential for nutrient deficiencies and constipation; avoid with SGLT2 inhibitors [152]. - Some patients can develop elevated LDL [179,187]. - Talk to patients about consuming healthy fats, plant proteins, and lower-carbohydrate sources of fiber [188,189]. - Unclear association with mortality. - Ensure adequate hydration and sodium and potassium balance [190].
Low-fat	- <30% of calories from fat. - Examples include DASH [164], AHA, Ornish-style, and some vegan diets [191,192].	- Lowers CVD risk, BP, and LDL; improves insulin resistance [164].	- Counsel not to replace animal foods with refined carbohydrate intake from ultra-processed food and sugar [187]. - Counsel on risk of low protein and iron intake if not planned well [137]. - Counsel to increase fiber and water [71].
High-protein	- 1.2–1.6 g/kg/day; mostly 20–35% of calories from protein [141,143,193].	- Preserves lean mass [136,143,158,194,195]. - Supports satiety and may assist with better long-term adherence due to influence on gut hormone signaling [193].	- May not be appropriate for CKD [150,193]. - Potential GI side effects [196]. - Adherence to high levels of protein may be challenging. - Counsel on lean and plant-based sources of protein, poultry, and fish, and to reduce processed meats [162]. - Ensure protein supplements such as bars and shakes minimize added sugar.
Very-low-calorie diet (VLCD)	- 800–1000 kcal/day; usually pre-packaged meals or shakes [53,179]. - Often low fat and high protein, some may provide ketogenic approach [196,197]. - Meant for short-term weight loss [179]. - A transition phase is usually included to assist in change to a normal diet [198].	- Rapid initial weight loss; improves glucose and BP quickly. - May induce diabetes remission [198,199].	- Requires medical supervision; risk of nutrient deficiency, gallstones, electrolyte abnormalities, severe fatigue, and arrhythmias [53,179]. - May be cost prohibitive. - Recommended to include long-term weight management strategies [179].
Time-restricted eating (TRE) and fasting	- Eating limited to 6–12 h/day (e.g., 12 pm–8 pm).	- May improve insulin sensitivity and adherence for some patients [200,201]. - Low cost and no need for tracking [202].	- Results may reflect overall calorie reduction [200,202]. - Mixed long-term data. - Avoid with SGLT-2 inhibitors [203]. - Adjust insulin and insulin secretagogues [152,204,205].

## Abbreviations

The following abbreviations are used in this manuscript:

ADLs	Activities of Daily Living
AHEAD	Action for Health in Diabetes
OM	Obesity Medication
BMI	Body Mass Index
CBTi	Cognitive Behavioral Therapy for Insomnia
CCM	Chronic Care Management
CDC	Centers for Disease Control and Prevention
CMS	Centers for Medicare & Medicaid Services
CNS	Central Nervous System
CoCM	Collaborative Care Management
CPT	Current Procedural Terminology
DASH	Dietary Approaches to Stop Hypertension
DIRECT	Diabetes Remission Clinical Trial
DKA	Diabetic Ketoacidosis
DO	Doctor of Osteopathic Medicine
DPP	Diabetes Prevention Program
DPPOS	Diabetes Prevention Program Outcomes Study
EC	Eigenvector Continuation
E&M	Evaluation and Management
FDA	U.S. Food and Drug Administration
FMD	Fasting Mimicking Diet
FODMAP	Fermentable Oligosaccharides, Disaccharides, Monosaccharides, and Polyols
GLP-1 RA	Glucagon-Like Peptide-1 Receptor Agonists
HAES	Health At Every Size
HDL	High-Density Lipoprotein
IBS	Irritable Bowel Syndrome
IBT	Intensive Behavioral Therapy
LDL	Low-Density Lipoprotein
LM	Lifestyle Medicine
MBS	Metabolic Bariatric Surgery
MBSAQIP	Metabolic and Bariatric Surgery Accreditation and Quality Improvement Program
MDT	Multidisciplinary Team
MI	Motivational Interviewing
MIND	Mediterranean-DASH Intervention for Neurogenerative Delay
MNT	Medical Nutrition Therapy
NEAT	Non-exercise Activity Thermogenesis
NCD-RisC	Non-communicable Disease Risk Factor Collaboration
NP	Nurse Practitioner
OMAD	One Meal A Day
OT	Occupational Therapist
PA	Physician Assistant
PREVIEW	Prevention of Diabetes Through Lifestyle Intervention and Population Studies in Europe and Around the World
PT	Physical Therapist
RCT	Randomized Controlled Trial
RDN	Registered Dietitian Nutritionist
RPM	Remote Patient Monitoring
RYGB	Roux-en Y Gastric Bypass
SLIM	Supervised Lifestyle and Integrative Medicine
SMA	Shared Medical Appointment
STEP	Semaglutide Treatment Effect in People with Obesity
TRE	Time-restricted Eating
USPSTF	United States Preventative Services Task Force
VLCD	Very-low-calorie Diet
WFPB	Whole Food Plant-based

## References

[1] Emmerich S., Fryar C., Stierman B., Ogden C. (2024). Obesity and Severe Obesity Prevalence in Adults: United States, August 2021–August 2023.

[2] World Health Organization Obesity and Overweight—Key Facts. https://www.who.int/news-room/fact-sheets/detail/obesity-and-overweight.

[3] Phelps N.H., Singleton R.K., Zhou B., Heap R.A., Mishra A., Bennett J.E., Paciorek C.J., Lhoste V.P., Carrillo-Larco R.M., Stevens G.A. (2024). Worldwide trends in underweight and obesity from 1990 to 2022: A pooled analysis of 3663 population-representative studies with 222 million children, adolescents, and adults. Lancet.

[4] Rubino F., Cummings D.E., Eckel R.H., Cohen R.V., Wilding J.P.H., Brown W.A., Stanford F.C., Batterham R.L., Farooqi I.S., Farpour-Lambert N.J. (2025). Definition and diagnostic criteria of clinical obesity. Lancet Diabetes Endocrinol..

[5] Bays H. (2014). Adiposopathy, “Sick Fat,” Ockham’s Razor, and Resolution of the Obesity Paradox. Curr. Atheroscler. Rep..

[6] Schwartz M.W., Seeley R.J., Zeltser L.M., Drewnowski A., Ravussin E., Redman L.M., Leibel R.L. (2017). Obesity Pathogenesis: An Endocrine Society Scientific Statement. Endocr. Rev..

[7] Lippman D., Stump M., Veazey E., Guimarães S.T., Rosenfeld R., Kelly J.H., Ornish D., Katz D.L. (2024). Foundations of Lifestyle Medicine and its Evolution. Mayo Clin. Proc. Innov. Qual. Outcomes.

[8] Frates B. (2023). The Power and Connection of the Six Pillars. Am. J. Lifestyle Med..

[9] Gigliotti L., Warshaw H., Evert A., Dawkins C., Schwartz J., Susie C., Kushner R., Subramanian S., Handu D., Rozga M. (2025). Incretin-Based Therapies and Lifestyle Interventions: The Evolving Role of Registered Dietitian Nutritionists in Obesity Care. J. Acad. Nutr. Diet..

[10] American College of Lifestyle Medicine (2023). American College of Lifestyle Medicine Official Position on Obesity and Overweight. https://lifestylemedicine.org/positions/.

[11] Golovaty I., Hagan S. (2025). Lifestyle Intervention Requirements for Novel Antiobesity Medications—Necessary Adjunct or Harmful Gatekeeper?. JAMA Intern. Med..

[12] Jensen M.D., Ryan D.H., Apovian C.M., Ard J.D., Comuzzie A.G., Donato K.A., Hu F.B., Hubbard V.S., Jakicic J.M., Kushner R.F. (2014). 2013 AHA/ACC/TOS Guideline for the Management of Overweight and Obesity in Adults: A Report of the American College of Cardiology/American Heart Association Task Force on Practice Guidelines and The Obesity Society. J. Am. Coll. Cardiol..

[13] Bays H., McCarthy W., Burridge K., Tondt J., Karjoo S., Christensen S., Ng J., Golden A., Davisson L., Richardson L. (2021). Obesity Algorithm eBook, Presented by the Obesity Medicine Association. https://obesitymedicine.org/resources/obesity-algorithm/.

[14] Grunvald E., Shah R., Hernaez R., Chandar A.K., Pickett-Blakely O., Teigen L.M., Harindhanavudhi T., Sultan S., Singh S., Davitkov P. (2022). AGA clinical practice guideline on pharmacological interventions for adults with obesity. Gastroenterology.

[15] Garvey W.T., Mechanick J.I., Brett E.M., Garber A.J., Hurley D.L., Jastreboff A.M., Nadolsky K., Pessah-Pollack R., Plodkowski R. (2016). American association of clinical endocrinologists and american college of endocrinology comprehensive clinical practice guidelines for medical care of patients with obesity. Endocr. Pract..

[16] Qi L., Heianza Y., Li X., Sacks F.M., Bray G.A. (2023). Toward precision weight-loss dietary interventions: Findings from the POUNDS lost trial. Nutrients.

[17] Heymsfield S.B., Wadden T.A. (2017). Mechanisms, Pathophysiology, and Management of Obesity. N. Engl. J. Med..

[18] Hauser M.E., Hartle J.C., Landry M.J., Fielding-Singh P., Shih C.W., Qin F., Rigdon J., Gardner C.D. (2024). Association of dietary adherence and dietary quality with weight loss success among those following low-carbohydrate and low-fat diets: A secondary analysis of the DIETFITS randomized clinical trial. Am. J. Clin. Nutr..

[19] Curry S.J., Krist A.H., Owens D.K., Barry M.J., Caughey A.B., Davidson K.W., Doubeni C.A., Epling J.W., Grossman D.C., Kemper A.R. (2018). Behavioral Weight Loss Interventions to Prevent Obesity-Related Morbidity and Mortality in Adults: US Preventive Services Task Force Recommendation Statement. JAMA.

[20] US Preventive Services Task Force (2020). Behavioral Counseling Interventions to Promote a Healthy Diet and Physical Activity for Cardiovascular Disease Prevention in Adults with Cardiovascular Risk Factors: US Preventive Services Task Force Recommendation Statement. JAMA.

[21] US Preventive Services Task Force (2021). Behavioral Counseling Interventions for Healthy Weight and Weight Gain in Pregnancy: US Preventive Services Task Force Recommendation Statement. JAMA.

[22] US Preventive Services Task Force (2024). Interventions for High Body Mass Index in Children and Adolescents: US Preventive Services Task Force Recommendation Statement. JAMA.

[23] US Preventive Services Task Force (2021). Screening for Prediabetes and Type 2 Diabetes: US Preventive Services Task Force Recommendation Statement. JAMA.

[24] Richman I., Rittenberg E. (2025). Lifestyle Modification for Obesity Management—A Cornerstone and Not a Roadblock. JAMA Intern. Med..

[25] Greaves C.J., Sheppard K.E., Abraham C., Hardeman W., Roden M., Evans P.H., Schwarz P., The IMAGE Study Group (2011). Systematic review of reviews of intervention components associated with increased effectiveness in dietary and physical activity interventions. BMC Public Health.

[26] Camacho P.M., Petak S.M., Binkley N., Diab D.L., Eldeiry L.S., Farooki A., Harris S.T., Hurley D.L., Kelly J., Lewiecki E.M. (2020). American Association of Clinical Endocrinologists/American College of Endocrinology Clinical Practice Guidelines for the Diagnosis and Treatment of Postmenopausal Osteoporosis-2020 Update. Endocr. Pract. Off. J. Am. Coll. Endocrinol. Am. Assoc. Clin. Endocrinol..

[27] Bauer K., Lau T., Schwille-Kiuntke J., Schild S., Hauner H., Stengel A., Zipfel S., Mack I. (2020). Conventional weight loss interventions across the different BMI obesity classes: A systematic review and quantitative comparative analysis. Eur. Eat. Disord. Rev..

[28] Farhana A., Rehman A. (2025). Metabolic Consequences of Weight Reduction. StatPearls.

[29] Machado A.M., Guimarães N.S., Bocardi V.B., da Silva T.P.R., do Carmo A.S., de Menezes M.C., Duarte C.K. (2022). Understanding weight regain after a nutritional weight loss intervention: Systematic review and meta-analysis. Clin. Nutr. ESPEN.

[30] Fothergill E., Guo J., Howard L., Kerns J.C., Knuth N.D., Brychta R., Chen K.Y., Skarulis M.C., Walter M., Walter P.J. (2016). Persistent metabolic adaptation 6 years after “The Biggest Loser” competition. Obesity.

[31] Thom G., Dombrowski S.U., Brosnahan N., Algindan Y.Y., Rosario Lopez-Gonzalez M., Roditi G., Lean M.E.J., Malkova D. (2020). The role of appetite-related hormones, adaptive thermogenesis, perceived hunger and stress in long-term weight-loss maintenance: A mixed-methods study. Eur. J. Clin. Nutr..

[32] Melby C.L., Paris H.L., Foright R.M., Peth J. (2017). Attenuating the Biologic Drive for Weight Regain Following Weight Loss: Must What Goes Down Always Go Back Up?. Nutrients.

[33] Montani J.-P., Schutz Y., Dulloo A.G. (2015). Dieting and weight cycling as risk factors for cardiometabolic diseases: Who is really at risk?. Obes. Rev..

[34] Park Y., Dodd K.W., Kipnis V., Thompson F.E., Potischman N., Schoeller D.A., Baer D.J., Midthune D., Troiano R.P., Bowles H. (2018). Comparison of self-reported dietary intakes from the Automated Self-Administered 24-h recall, 4-d food records, and food-frequency questionnaires against recovery biomarkers. Am. J. Clin. Nutr..

[35] Vadiveloo M., Lichtenstein A.H., Anderson C., Aspry K., Foraker R., Griggs S., Hayman L.L., Johnston E., Stone N.J., Thorndike A.N. (2020). Rapid Diet Assessment Screening Tools for Cardiovascular Disease Risk Reduction Across Healthcare Settings: A Scientific Statement From the American Heart Association. Circ. Cardiovasc. Qual. Outcomes.

[36] Gans K.M., Ross E., Barner C.W., Wylie-Rosett J., McMurray J., Eaton C. (2003). REAP and WAVE: New tools to rapidly assess/discuss nutrition with patients. J. Nutr..

[37] Lara-Breitinger K.M., Medina Inojosa J.R., Li Z., Kunzova S., Lerman A., Kopecky S.L., Lopez-Jimenez F. (2023). Validation of a Brief Dietary Questionnaire for Use in Clinical Practice: Mini-EAT (Eating Assessment Tool). J. Am. Heart Assoc..

[38] Kronsteiner-Gicevic S., Tello M., Lincoln L.E., Kondo J.K., Naidoo U., Fung T.T., Willett W.C., Thorndike A.N. (2023). Validation of the Rapid Prime Diet Quality Score Screener (rPDQS), A Brief Dietary Assessment Tool with Simple Traffic Light Scoring. J. Acad. Nutr. Diet..

[39] Karlsen M.C., Staffier K.L., Pollard K.J., Cara K.C., Hulit S.M., Campbell E.K., Friedman S.M. (2024). Piloting a brief assessment to capture consumption of whole plant food and water: Version 1.0 of the American College of Lifestyle Medicine Diet Screener (ACLM Diet Screener). Front. Nutr..

[40] Georgoulis M., Yiannakouris N., Kechribari I., Lamprou K., Perraki E., Vagiakis E., Kontogianni M.D. (2022). Dose-response relationship between weight loss and improvements in obstructive sleep apnea severity after a diet/lifestyle interventions: Secondary analyses of the “MIMOSA” randomized clinical trial. J. Clin. Sleep Med..

[41] Mukherjee S., Patel S.R., Kales S.N., Ayas N.T., Strohl K.P., Gozal D., Malhotra A., American Thoracic Society ad hoc Committee on Healthy Sleep (2015). An Official American Thoracic Society Statement: The Importance of Healthy Sleep. Recommendations and Future Priorities. Am. J. Respir. Crit. Care Med..

[42] Morin C.M., Buysse D.J. (2024). Management of Insomnia. N. Engl. J. Med..

[43] Aboona M.B., Danpanichkul P., Chen V.L., Rangan P., Kim D., Alkhouri N., Fallon M.B., Noureddin M., Arab J.P., Wijarnpreecha K. (2024). Mortality outcomes in individuals with MASLD versus MASLD and increased alcohol intake. J. Gastroenterol. Hepatol..

[44] Pangalangan J., Puma J., Tollefson M., Frates B. (2024). Development and Psychometric Evaluation of the Lifestyle Medicine Health Behavior Scale. Am. J. Lifestyle Med..

[45] Bonnet J.P. (2024). Content and Face Validation of the Lifestyle Medicine Assessment. Am. J. Lifestyle Med..

[46] Rozanski A., Sakul S., Narula J., Berman D. (2023). Assessment of lifestyle “vital signs” in healthcare settings. Prog. Cardiovasc. Dis..

[47] Mechley A.R., Dysinger W. (2015). Intensive Therapeutic Lifestyle Change Programs: A Progressive Way to Successfully Manage Health Care. Am. J. Lifestyle Med..

[48] The Diabetes Prevention Program Research Group (2002). Reduction in the Incidence of Type 2 Diabetes with Lifestyle Intervention or Metformin. N. Engl. J. Med..

[49] Center for Medicare and Medicaid Services National Coverage Determination—Intensive Behavioral Therapy for Obesity (210.12). Medicare Coverage Database. https://www.cms.gov/medicare-coverage-database/view/ncd.aspx?NCDId=353.

[50] Chao A.M., Moore M., Wadden T.A. (2025). The past, present, and future of behavioral obesity treatment. Int. J. Obes..

[51] Wadden T.A., Tsai A.G., Tronieri J.S. (2019). A Protocol to Deliver Intensive Behavioral Therapy (IBT) for Obesity in Primary Care Settings: The MODEL-IBT Program. Obesity.

[52] Elmaleh-Sachs A., Schwartz J.L., Bramante C.T., Nicklas J.M., Gudzune K.A., Jay M. (2023). Obesity Management in Adults: A Review. JAMA.

[53] American Diabetes Association Professional Practice Committee (2024). 8. Obesity and Weight Management for the Prevention and Treatment of Type 2 Diabetes: Standards of Care in Diabetes–2025. Diabetes Care.

[54] Nadolsky K., Addison B., Agarwal M., Almandoz J.P., Bird M.D., Chaplin M.D., Garvey W.T., Kyle T.K. (2023). American Association of Clinical Endocrinology Consensus Statement: Addressing Stigma and Bias in the Diagnosis and Management of Patients with Obesity/Adiposity-Based Chronic Disease and Assessing Bias and Stigmatization as Determinants of Disease Severity. Endocr. Pract..

[55] Puhl R.M. (2023). Weight Stigma and Barriers to Effective Obesity Care. Gastroenterol. Clin..

[56] Talumaa B., Brown A., Batterham R.L., Kalea A.Z. (2022). Effective strategies in ending weight stigma in healthcare. Obes. Rev..

[57] Eggerichs L.A., Wilson O.W.A., Chaplin J.E., Ramos Salas X. (2024). Weight Stigma in Latin America, Asia, the Middle East, and Africa: A Scoping Review. Obes. Facts.

[58] Althumiri N.A., Basyouni M.H., AlMousa N., AlJuwaysim M.F., Alhamdan A.A., Al-Qahtani F.S., BinDhim N.F., Alqahtani S.A. (2021). Exploring weight stigma in Saudi Arabia: A nationwide cross-sectional study. Int. J. Environ. Res. Public. Health.

[59] Brenton-Peters J., Consedine N.S., Boggiss A., Wallace-Boyd K., Roy R., Serlachius A. (2021). Self-compassion in weight management: A systematic review. J. Psychosom. Res..

[60] Braun T.D., Olson K., Panza E., Lillis J., Schumacher L., Abrantes A.M., Kunicki Z., Unick J.L. (2022). Internalized weight stigma in women with class III obesity: A randomized controlled trial of a virtual lifestyle modification intervention followed by a mindful self-compassion intervention. Obes. Sci. Pract..

[61] Thedinga H.K., Zehl R., Thiel A. (2021). Weight stigma experiences and self-exclusion from sport and exercise settings among people with obesity. BMC Public Health.

[62] Pickett A.C., and Cunningham G.B. (2017). Physical Activity for Every Body: A Model for Managing Weight Stigma and Creating Body-Inclusive Spaces. Quest.

[63] Lima Fogaca J., Zuest L., Lee S., Squires N., Clifford D. (2024). Weight Inclusive Thinking for Fitness Spaces (WIT FITS): A Three-Month Follow-Up of a Weight Stigma Intervention for Exercise Professionals. Recreat. Sports J..

[64] Greenleaf C., Rodriguez A.M. (2021). Living in a Larger Body: Do Exercise Motives Influence Associations between Body Image and Exercise Avoidance Motivation?. Int. J. Environ. Res. Public. Health.

[65] Cox A.E., Ullrich-French S., Tylka T.L., McMahon A.K. (2019). The roles of self-compassion, body surveillance, and body appreciation in predicting intrinsic motivation for physical activity: Cross-sectional associations, and prospective changes within a yoga context. Body Image.

[66] Wood M., Pila E. (2022). Investigating the effects of fit-normative and weight-inclusive Instagram images on women’s exercise motivations. Body Image.

[67] Tylka T.L., Calogero R.M., Daníelsdóttir S. (2020). Intuitive eating is connected to self-reported weight stability in community women and men. Eat. Disord..

[68] Satter E. (2007). Eating Competence: Definition and Evidence for the Satter Eating Competence Model. J. Nutr. Educ. Behav..

[69] Psota T.L., Lohse B., West S.G. (2007). Associations between Eating Competence and Cardiovascular Disease Biomarkers. J. Nutr. Educ. Behav..

[70] Mensinger J.L., Calogero R.M., Stranges S., Tylka T.L. (2016). A weight-neutral versus weight-loss approach for health promotion in women with high BMI: A randomized-controlled trial. Appetite.

[71] Zuraikat F.M., Rolls B.J. (2023). Dietary Energy Density and Its Contribution to Weight Control. Handbook of Obesity-Volume 2.

[72] Rolls B.J. (2017). Dietary energy density: Applying behavioural science to weight management. Nutr. Bull..

[73] Da Luz F.Q., Hay P., Touyz S., Sainsbury A. (2018). Obesity with Comorbid Eating Disorders: Associated Health Risks and Treatment Approaches. Nutrients.

[74] Yoon C., Mason S.M., Hooper L., Eisenberg M.E., Neumark-Sztainer D. (2020). Disordered Eating Behaviors and 15-year Trajectories in Body Mass Index: Findings From Project Eating and Activity in Teens and Young Adults (EAT). J. Adolesc. Health.

[75] Xu Y., Echouffo Tcheugui J.B., Coresh J., Grams M.E., Selvin E., Fang M., Shin J.-I. (2025). Trends in obesity and glucagon-like peptide-1 receptor agonist prescriptions in type 1 diabetes in the United States. Diabetes Obes. Metab..

[76] Ladebo L., Ernst M.T., Mailhac A., Dirksen C., Bojsen-Møller K.N., Pottegård A. (2024). Real-World Use of Semaglutide for Weight Management: Patient Characteristics and Dose Titration-A Danish Cohort Study. Diabetes Care.

[77] Le Roux C.W., Koroleva A., Larsen S., Foot E. (2025). Anti-obesity treatment preferences of healthcare providers and people living with obesity: A survey-based study. Clin. Obes..

[78] Blundell J., Finlayson G., Axelsen M., Flint A., Gibbons C., Kvist T., Hjerpsted J.B. (2017). Effects of once-weekly semaglutide on appetite, energy intake, control of eating, food preference and body weight in subjects with obesity. Diabetes Obes. Metab..

[79] Jastreboff A.M., Aronne L.J., Ahmad N.N., Wharton S., Connery L., Alves B., Kiyosue A., Zhang S., Liu B., Bunck M.C. (2022). Tirzepatide Once Weekly for the Treatment of Obesity. N. Engl. J. Med..

[80] Wadden T.A., Bailey T.S., Billings L.K., Davies M., Frias J.P., Koroleva A., Lingvay I., O’Neil P.M., Rubino D.M., Skovgaard D. (2021). Effect of Subcutaneous Semaglutide vs Placebo as an Adjunct to Intensive Behavioral Therapy on Body Weight in Adults With Overweight or Obesity: The STEP 3 Randomized Clinical Trial. JAMA.

[81] Khan S.S., Ndumele C.E., Kazi D.S. (2025). Discontinuation of Glucagon-Like Peptide-1 Receptor Agonists. JAMA.

[82] Highlights of Prescribing Information: Wegovy (Semaglutide) Injection, for Subcutaneous Use Initial Approval, 2017. https://www.accessdata.fda.gov/drugsatfda_docs/label/2023/215256s007lbl.pdf.

[83] Office of the Commissioner FDA Approves New Medication for Chronic Weight Management. FDA. https://www.fda.gov/news-events/press-announcements/fda-approves-new-medication-chronic-weight-management.

[84] Ard J., Cannon A., Lewis C.E., Lofton H., Vang Skjøth T., Stevenin B., Pi-Sunyer X. (2016). Efficacy and safety of liraglutide 3.0 mg for weight management are similar across races: Subgroup analysis across the SCALE and phase II randomized trials. Diabetes Obes. Metab..

[85] Wadden T.A., Chao A.M., Machineni S., Kushner R., Ard J., Srivastava G., Halpern B., Zhang S., Chen J., Bunck M.C. (2023). Tirzepatide after intensive lifestyle intervention in adults with overweight or obesity: The SURMOUNT-3 phase 3 trial. Nat. Med..

[86] Das S.R., Everett B.M., Birtcher K.K., Brown J.M., Januzzi J.L., Kalyani R.R., Kosiborod M., Magwire M., Morris P.B., Neumiller J.J. (2020). 2020 Expert Consensus Decision Pathway on Novel Therapies for Cardiovascular Risk Reduction in Patients With Type 2 Diabetes. J. Am. Coll. Cardiol..

[87] Gudzune K.A., Kushner R.F. (2024). Medications for Obesity: A Review. JAMA.

[88] Karakasis P., Patoulias D., Fragakis N., Mantzoros C.S. (2025). Effect of glucagon-like peptide-1 receptor agonists and co-agonists on body composition: Systematic review and network meta-analysis. Metabolism.

[89] Jiao R., Lin C., Cai X., Wang J., Wang Y., Lv F., Yang W., Ji L. (2025). Characterizing body composition modifying effects of a glucagon-like peptide 1 receptor-based agonist: A meta-analysis. Diabetes Obes. Metab..

[90] Linge J., Birkenfeld A.L., Neeland I.J. (2024). Muscle Mass and Glucagon-Like Peptide-1 Receptor Agonists: Adaptive or Maladaptive Response to Weight Loss?. Circulation.

[91] Mechanick J.I., Butsch W.S., Christensen S.M., Hamdy O., Li Z., Prado C.M., Heymsfield S.B. (2025). Strategies for minimizing muscle loss during use of incretin-mimetic drugs for treatment of obesity. Obes. Rev. Off. J. Int. Assoc. Study Obes..

[92] Lundgren J.R., Janus C., Jensen S.B.K., Juhl C.R., Olsen L.M., Christensen R.M., Svane M.S., Bandholm T., Bojsen-Møller K.N., Blond M.B. (2021). Healthy Weight Loss Maintenance with Exercise, Liraglutide, or Both Combined. N. Engl. J. Med..

[93] Beavers K.M., Cortes T.M., Foy C.M., Dinkla L., Reyes San Martin F., Ard J.D., Serra M.C., Beavers D.P. (2025). GLP1Ra-based therapies and DXA-acquired musculoskeletal health outcomes: A focused meta-analysis of placebo-controlled trials. Obesity.

[94] Seimon R.V., Wild-Taylor A.L., Keating S.E., McClintock S., Harper C., Gibson A.A., Johnson N.A., Fernando H.A., Markovic T.P., Center J.R. (2019). Effect of Weight Loss via Severe vs Moderate Energy Restriction on Lean Mass and Body Composition Among Postmenopausal Women with Obesity: The TEMPO Diet Randomized Clinical Trial. JAMA Netw. Open.

[95] Turicchi J., O’Driscoll R., Finlayson G., Beaulieu K., Deighton K., Stubbs R.J. (2019). Associations between the rate, amount, and composition of weight loss as predictors of spontaneous weight regain in adults achieving clinically significant weight loss: A systematic review and meta-regression. Obes. Rev..

[96] Roust L.R., DiBaise J.K. (2017). Nutrient deficiencies prior to bariatric surgery. Curr. Opin. Clin. Nutr. Metab. Care.

[97] Astrup A., Bügel S. (2019). Overfed but undernourished: Recognizing nutritional inadequacies/deficiencies in patients with overweight or obesity. Int. J. Obes..

[98] Poli V.F.S., Sanches R.B., dos Santos Moraes A., Fidalgo J.P.N., Nascimento M.A., Bresciani P., Andrade-Silva S.G., Cipullo M.A.T., Clemente J.C., Caranti D.A. (2017). The excessive caloric intake and micronutrient deficiencies related to obesity after a long-term interdisciplinary therapy. Nutrition.

[99] Fulgoni V.L., Agler A., Ricciuto L., DiFrancesco L., Williams D., Hertzler S.R. (2024). Impact of Simulated Caloric Reduction on Nutrient Adequacy Among U.S. Adults with Overweight or Obesity (National Health and Nutrition Examination Survey [NHANES] 2015–2018). J. Nutr..

[100] Chang L., Chey W.D., Imdad A., Almario C.V., Bharucha A.E., Diem S., Greer K.B., Hanson B., Harris L.A., Ko C. (2023). American Gastroenterological Association-American College of Gastroenterology clinical practice guideline: Pharmacological management of chronic idiopathic constipation. Gastroenterology.

[101] Gentinetta S., Sottotetti F., Manuelli M., Cena H. (2024). Dietary Recommendations for the Management of Gastrointestinal Symptoms in Patients Treated with GLP-1 Receptor Agonist. Diabetes Metab. Syndr. Obes..

[102] Harvie R.M., Chisholm A.W., Bisanz J.E., Burton J.P., Herbison P., Schultz K., Schultz M. (2017). Long-term irritable bowel syndrome symptom control with reintroduction of selected FODMAPs. World J. Gastroenterol..

[103] US Food and Drug Administration Contrave—HIGHLIGHTS OF PRESCRIBING INFORMATION. https://www.accessdata.fda.gov/drugsatfda_docs/label/2024/200063s022lbl.pdf.

[104] He L., Li Q., Yang Y., Li J., Luo W., Huang Y., Zhong X. (2024). Pharmacovigilance study of GLP-1 receptor agonists for metabolic and nutritional adverse events. Front. Pharmacol..

[105] Winzeler B., Sailer C.O., Coynel D., Zanchi D., Vogt D.R., Urwyler S.A., Refardt J., Christ-Crain M. (2021). A randomized controlled trial of the GLP-1 receptor agonist dulaglutide in primary polydipsia. J. Clin. Investig..

[106] US Food and Drug Administration Topiramate: Highlights of Prescribing Information. May 2017. https://www.accessdata.fda.gov/drugsatfda_docs/label/2017/020505s057_020844s048lbl.pdf.

[107] Alhomoud I.S., Cook E., Patel D., Brown R.E., Dixon D.L. (2024). Effect of pharmacist interventions on the management of overweight and obesity: A systematic review. J. Am. Pharm. Assoc..

[108] Mirza N., Marson A.G., Pirmohamed M. (2009). Effect of topiramate on acid–base balance: Extent, mechanism and effects. Br. J. Clin. Pharmacol..

[109] Eisenberg D., Shikora S.A., Aarts E., Aminian A., Angrisani L., Cohen R.V., Luca M.D., Faria S.L., Goodpaster K.P.S., Haddad A. (2022). 2022 American Society for Metabolic and Bariatric Surgery (ASMBS) and International Federation for the Surgery of Obesity and Metabolic Disorders (IFSO): Indications for Metabolic and Bariatric Surgery. Surg. Obes. Relat. Dis..

[110] Arterburn D.E., Telem D.A., Kushner R.F., Courcoulas A.P. (2020). Benefits and Risks of Bariatric Surgery in Adults: A Review. JAMA.

[111] Courcoulas A.P., Daigle C.R., Arterburn D.E. (2023). Long term outcomes of metabolic/bariatric surgery in adults. BMJ.

[112] Wiebe N., Tonelli M. (2024). Long-term clinical outcomes of bariatric surgery in adults with severe obesity: A population-based retrospective cohort study. PLoS ONE.

[113] Al-Khyatt W., Ryall R., Leeder P., Ahmed J., Awad S. (2017). Predictors of Inadequate Weight Loss After Laparoscopic Gastric Bypass for Morbid Obesity. Obes. Surg..

[114] Anderin C., Gustafsson U.O., Heijbel N., Thorell A. (2015). Weight Loss Before Bariatric Surgery and Postoperative Complications: Data From the Scandinavian Obesity Registry (SOReg). Ann. Surg..

[115] Mechanick J.I., Apovian C., Brethauer S., Garvey W.T., Joffe A.M., Kim J., Kushner R.F., Lindquist R., Pessah-Pollack R., Seger J. (2020). Clinical practice guidelines for the perioperative nutrition, metabolic, and nonsurgical support of patients undergoing bariatric procedures—2019 update: Cosponsored by American Association of Clinical Endocrinologists/American College of Endocrinology, The Obesity Society, American Society for Metabolic & Bariatric Surgery, Obesity Medicine Association, and American Society of Anesthesiologists. Surg. Obes. Relat. Dis..

[116] Carter J., Chang J., Birriel T.J., Moustarah F., Sogg S., Goodpaster K., Benson-Davies S., Chapmon K., Eisenberg D. (2021). ASMBS position statement on preoperative patient optimization before metabolic and bariatric surgery. Surg. Obes. Relat. Dis. Off. J. Am. Soc. Bariatr. Surg..

[117] Athanasiadis D.I., Martin A., Kapsampelis P., Monfared S., Stefanidis D. (2021). Factors associated with weight regain post-bariatric surgery: A systematic review. Surg. Endosc..

[118] Sherf Dagan S., Goldenshluger A., Globus I., Schweiger C., Kessler Y., Kowen Sandbank G., Ben-Porat T., Sinai T. (2017). Nutritional Recommendations for Adult Bariatric Surgery Patients: Clinical Practice. Adv. Nutr..

[119] Stocker R., Ceyhan M., Schönenberger K.A., Stanga Z., Reber E. (2022). Nutrient and fluid requirements in post-bariatric patients performing physical activity: A systematic review. Nutrition.

[120] Kumbhari V., Cummings D.E., Kalloo A.N., Schauer P.R. (2021). AGA Clinical Practice Update on Evaluation and Management of Early Complications After Bariatric/Metabolic Surgery: Expert Review. Clin. Gastroenterol. Hepatol..

[121] Hui C., Bauza G.J. (2025). Dumping Syndrome. StatPearls.

[122] Mages M., Shojaa M., Kohl M., von Stengel S., Becker C., Gosch M., Jakob F., Kerschan-Schindl K., Kladny B., Klöckner N. (2021). Exercise Effects on Bone Mineral Density in Men. Nutrients.

[123] Kemmler W., Shojaa M., Kohl M., von Stengel S. (2020). Effects of Different Types of Exercise on Bone Mineral Density in Postmenopausal Women: A Systematic Review and Meta-analysis. Calcif. Tissue Int..

[124] Sackner-Bernstein J., Kanter D., Kaul S. (2015). Dietary intervention for overweight and obese adults: Comparison of low-carbohydrate and low-fat diets. A meta-analysis. PLoS ONE.

[125] Karagun B., Baklaci N. (2024). Comparative analysis of basal metabolic rate measurement methods in overweight and obese individuals: A retrospective study. Medicine.

[126] Anderberg I., Kemps E., Prichard I. (2025). The link between the use of diet and fitness monitoring apps, body image and disordered eating symptomology: A systematic review. Body Image.

[127] Hahn S.L., Bornstein C., Burnette C.B., Loth K.A., Neumark-Sztainer D. (2024). A mixed-methods longitudinal examination of weight-related self-monitoring and disordered eating among a population-based sample of emerging adults. J. Eat. Disord..

[128] Turner-McGrievy G.M., Yang C.-H., Monroe C., Pellegrini C., West D.S. (2021). Is Burden Always Bad? Emerging Low-Burden Approaches to Mobile Dietary Self-monitoring and the Role Burden Plays with Engagement. J. Technol. Behav. Sci..

[129] Gardner B., Rebar A.L. (2019). Habit Formation and Behavior Change. Oxford Research Encyclopedia of Psychology.

[130] Churuangsuk C., Griffiths D., Lean M.E.J., Combet E. (2019). Impacts of carbohydrate-restricted diets on micronutrient intakes and status: A systematic review. Obes. Rev..

[131] Lapik I.A., Galchenko A.V., Gapparova K.M. (2020). Micronutrient status in obese patients: A narrative review. Obes. Med..

[132] Madanchi M., Fagagnini S., Fournier N., Biedermann L., Zeitz J., Battegay E., Zimmerli L., Vavricka S.R., Rogler G., Scharl M. (2018). The Relevance of Vitamin and Iron Deficiency in Patients with Inflammatory Bowel Diseases in Patients of the Swiss IBD Cohort. Inflamm. Bowel Dis..

[133] Reytor-González C., Frias-Toral E., Nuñez-Vásquez C., Parise-Vasco J.M., Zambrano-Villacres R., Simancas-Racines D., Schiavo L. (2025). Preventing and Managing Pre- and Postoperative Micronutrient Deficiencies: A Vital Component of Long-Term Success in Bariatric Surgery. Nutrients.

[134] Gasmi A., Bjørklund G., Mujawdiya P.K., Semenova Y., Peana M., Dosa A., Piscopo S., Gasmi Benahmed A., Costea D.O. (2022). Micronutrients deficiences in patients after bariatric surgery. Eur. J. Nutr..

[135] Bogataj Jontez N., Šik Novak K., Jenko Pražnikar Z., Petelin A., Kenig S., Mohorko N. (2024). Does Dietary Supplement Use Increase Micronutrient Intake Adequacy in Healthy Adults with Habitual Omnivorous, Vegetarian, Vegan, and Low-Carbohydrate High-Fat Diets?. Nutrients.

[136] Rogeri P.S., Zanella R., Martins G.L., Garcia M.D.A., Leite G., Lugaresi R., Gasparini S.O., Sperandio G.A., Ferreira L.H.B., Souza-Junior T.P. (2021). Strategies to Prevent Sarcopenia in the Aging Process: Role of Protein Intake and Exercise. Nutrients.

[137] Hansen T.T., Astrup A., Sjödin A. (2021). Are Dietary Proteins the Key to Successful Body Weight Management? A Systematic Review and Meta-Analysis of Studies Assessing Body Weight Outcomes after Interventions with Increased Dietary Protein. Nutrients.

[138] Volek J.S., Kackley M.L., Buga A. (2024). Nutritional Considerations During Major Weight Loss Therapy: Focus on Optimal Protein and a Low-Carbohydrate Dietary Pattern. Curr. Nutr. Rep..

[139] Wolfe R.R., Cifelli A.M., Kostas G., Kim I.-Y. (2017). Optimizing Protein Intake in Adults: Interpretation and Application of the Recommended Dietary Allowance Compared with the Acceptable Macronutrient Distribution Range. Adv. Nutr..

[140] Heymsfield S.B., Shapses S.A. (2024). Guidance on Energy and Macronutrients across the Life Span. N. Engl. J. Med..

[141] Leidy H.J., Clifton P.M., Astrup A., Wycherley T.P., Westerterp-Plantenga M.S., Luscombe-Marsh N.D., Woods S.C., Mattes R.D. (2015). The role of protein in weight loss and maintenance234. Am. J. Clin. Nutr..

[142] Mateo-Gallego R., Marco-Benedí V., Perez-Calahorra S., Bea A.M., Baila-Rueda L., Lamiquiz-Moneo I., de Castro-Orós I., Cenarro A., Civeira F. (2017). Energy-restricted, high-protein diets more effectively impact cardiometabolic profile in overweight and obese women than lower-protein diets. Clin. Nutr. Edinb. Scotl..

[143] Campbell W.W., Deutz N.E.P., Volpi E., Apovian C.M. (2023). Nutritional Interventions: Dietary Protein Needs and Influences on Skeletal Muscle of Older Adults. J. Gerontol. Ser. A.

[144] Hevia-Larraín V., Gualano B., Longobardi I., Gil S., Fernandes A.L., Costa L.A.R., Pereira R.M.R., Artioli G.G., Phillips S.M., Roschel H. (2021). High-Protein Plant-Based Diet Versus a Protein-Matched Omnivorous Diet to Support Resistance Training Adaptations: A Comparison Between Habitual Vegans and Omnivores. Sports Med..

[145] Jäger R., Kerksick C.M., Campbell B.I., Cribb P.J., Wells S.D., Skwiat T.M., Purpura M., Ziegenfuss T.N., Ferrando A.A., Arent S.M. (2017). International Society of Sports Nutrition Position Stand: Protein and exercise. J. Int. Soc. Sports Nutr..

[146] Thomas D.T., Erdman K.A., Burke L.M. (2016). Position of the Academy of Nutrition and Dietetics, Dietitians of Canada, and the American College of Sports Medicine: Nutrition and Athletic Performance. J. Acad. Nutr. Diet..

[147] Pang M.D., Goossens G.H., Blaak E.E. (2021). The Impact of Artificial Sweeteners on Body Weight Control and Glucose Homeostasis. Front. Nutr..

[148] Lenhart A., Chey W.D. (2017). A Systematic Review of the Effects of Polyols on Gastrointestinal Health and Irritable Bowel Syndrome. Adv. Nutr..

[149] American Diabetes Association Professional Practice Committee (2024). 11. Chronic Kidney Disease and Risk Management: Standards of Care in Diabetes—2025. Diabetes Care.

[150] Chang L.L., Rhee C.M., Kalantar-Zadeh K., Woodrow G. (2024). Dietary Protein Restriction in Patients with Chronic Kidney Disease. N. Engl. J. Med..

[151] Kalantar-Zadeh K., Fouque D. (2017). Nutritional Management of Chronic Kidney Disease. N. Engl. J. Med..

[152] American Diabetes Association Professional Practice Committee (2024). 5. Facilitating Positive Health Behaviors and Well-being to Improve Health Outcomes: Standards of Care in Diabetes—2025. Diabetes Care.

[153] The Diabetes Prevention Program Research Group (2009). 10-year follow-up of diabetes incidence and weight loss in the Diabetes Prevention Program Outcomes Study. Lancet.

[154] Qin W., Ying W., Hamaker B., Zhang G. (2021). Slow digestion-oriented dietary strategy to sustain the secretion of GLP-1 for improved glucose homeostasis. Compr. Rev. Food Sci. Food Saf..

[155] Luo K., Wang X., Zhang G. (2018). The anti-obesity effect of starch in a whole grain-like structural form. Food Funct..

[156] Rolls B.J. (2018). The role of portion size, energy density, and variety in obesity and weight management. Handbook of Obesity Treatment.

[157] Lieberman H.R., Fulgoni V.L., Agarwal S., Pasiakos S.M., Berryman C.E. (2020). Protein intake is more stable than carbohydrate or fat intake across various US demographic groups and international populations. Am. J. Clin. Nutr..

[158] Naseeb M.A., Volpe S.L. (2017). Protein and exercise in the prevention of sarcopenia and aging. Nutr. Res..

[159] Giglio B.M., Lobo P.C.B., Pimentel G.D. (2023). Effects of whey protein supplementation on adiposity, body weight, and glycemic parameters: A synthesis of evidence. Nutr. Metab. Cardiovasc. Dis..

[160] Kjølbæk L., Sørensen L.B., Søndertoft N.B., Rasmussen C.K., Lorenzen J.K., Serena A., Astrup A., Larsen L.H. (2017). Protein supplements after weight loss do not improve weight maintenance compared with recommended dietary protein intake despite beneficial effects on appetite sensation and energy expenditure: A randomized, controlled, double-blinded trial. Am. J. Clin. Nutr..

[161] Snetselaar L.G., de Jesus J.M., DeSilva D.M., Stoody E.E. (2021). Dietary Guidelines for Americans, 2020–2025: Understanding the Scientific Process, Guidelines, and Key Recommendations. Nutr. Today.

[162] Hoelscher D.M., Tobias D., Deierlein A., Gardner C., Giovannucci E., Anderson C.A.M., Booth S., Raynor H., Fung T., Stanford F.C. (2024). Dietary Patterns and Growth, Body Composition, and Risk of Obesity: A Systematic Review.

[163] McCarthy D., Berg A. (2021). Weight Loss Strategies and the Risk of Skeletal Muscle Mass Loss. Nutrients.

[164] Chiavaroli L., Viguiliouk E., Nishi S.K., Blanco Mejia S., Rahelić D., Kahleová H., Salas-Salvadó J., Kendall C.W., Sievenpiper J.L. (2019). DASH Dietary Pattern and Cardiometabolic Outcomes: An Umbrella Review of Systematic Reviews and Meta-Analyses. Nutrients.

[165] Estruch R., Ros E., Salas-Salvadó J., Covas M.-I., Corella D., Arós F., Gómez-Gracia E., Ruiz-Gutiérrez V., Fiol M., Lapetra J. (2018). Primary Prevention of Cardiovascular Disease with a Mediterranean Diet Supplemented with Extra-Virgin Olive Oil or Nuts. N. Engl. J. Med..

[166] Satija A., Bhupathiraju S.N., Rimm E.B., Spiegelman D., Chiuve S.E., Borgi L., Willett W.C., Manson J.E., Sun Q., Hu F.B. (2016). Plant-Based Dietary Patterns and Incidence of Type 2 Diabetes in US Men and Women: Results from Three Prospective Cohort Studies. PLoS Med..

[167] Satija A., Hu F.B. (2018). Plant-based diets and cardiovascular health. Trends Cardiovasc. Med..

[168] Rico-Campà A., Martínez-González M.A., Alvarez-Alvarez I., de Deus Mendonça R., De La Fuente-Arrillaga C., Gómez-Donoso C., Bes-Rastrollo M. (2019). Association between consumption of ultra-processed foods and all cause mortality: SUN prospective cohort study. BMJ.

[169] Srour B., Fezeu L.K., Kesse-Guyot E., Allès B., Méjean C., Andrianasolo R.M., Chazelas E., Deschasaux M., Hercberg S., Galan P. (2019). Ultra-processed food intake and risk of cardiovascular disease: Prospective cohort study (NutriNet-Santé). BMJ.

[170] Fardet A. (2016). Minimally processed foods are more satiating and less hyperglycemic than ultra-processed foods: A preliminary study with 98 ready-to-eat foods. Food Funct..

[171] Monteiro C.A., Cannon G., Levy R., Moubarac J.-C., Jaime P., Martins A.P., Canella D., Louzada M., Parra D. (2016). NOVA. The star shines bright. World Nutr..

[172] Martinez-Steele E., Khandpur N., Batis C., Bes-Rastrollo M., Bonaccio M., Cediel G., Huybrechts I., Juul F., Levy R.B., da Costa Louzada M.L. (2023). Best practices for applying the Nova food classification system. Nat. Food.

[173] Martínez Steele E., Popkin B.M., Swinburn B., Monteiro C.A. (2017). The share of ultra-processed foods and the overall nutritional quality of diets in the US: Evidence from a nationally representative cross-sectional study. Popul. Health Metr..

[174] Hall K.D. (2018). Ultra-Processed Diets Cause Excess Calorie Intake and Weight Gain: A One-Month Inpatient Randomized Controlled Trial of ad Libitum Food Intake. https://osf.io/preprints/nutrixiv/w3zh2/.

[175] Huang Y., Chen Z., Chen B., Li J., Yuan X., Li J., Wang W., Dai T., Chen H., Wang Y. (2023). Dietary sugar consumption and health: Umbrella review. BMJ.

[176] Hu H., Zhao Y., Feng Y., Yang X., Li Y., Wu Y., Yuan L., Zhang J., Li T., Huang H. (2023). Consumption of whole grains and refined grains and associated risk of cardiovascular disease events and all-cause mortality: A systematic review and dose-response meta-analysis of prospective cohort studies. Am. J. Clin. Nutr..

[177] Gaesser G.A. (2022). Refined Grain Intake and Risk of Type 2 Diabetes. Mayo Clin. Proc..

[178] Waddell I.S., Orfila C. (2023). Dietary fiber in the prevention of obesity and obesity-related chronic diseases: From epidemiological evidence to potential molecular mechanisms. Crit. Rev. Food Sci. Nutr..

[179] Alexander L., Christensen S.M., Richardson L., Ingersoll A.B., Burridge K., Golden A., Karjoo S., Cortez D., Shelver M., Bays H.E. (2022). Nutrition and physical activity: An Obesity Medicine Association (OMA) Clinical Practice Statement 2022. Obes. Pillars.

[180] Katz D.L., Doughty K.N., Geagan K., Jenkins D.A., Gardner C.D. (2019). Perspective: The Public Health Case for Modernizing the Definition of Protein Quality. Adv. Nutr..

[181] Kuo Y.-Y., Chang H.-Y., Huang Y.-C., Liu C.-W. (2022). Effect of Whey Protein Supplementation in Postmenopausal Women: A Systematic Review and Meta-Analysis. Nutrients.

[182] Gardner C.D., Trepanowski J.F., Del Gobbo L.C., Hauser M.E., Rigdon J., Ioannidis J.P., Desai M., King A.C. (2018). Effect of low-fat vs low-carbohydrate diet on 12-month weight loss in overweight adults and the association with genotype pattern or insulin secretion: The DIETFITS randomized clinical trial. JAMA.

[183] Konieczna J., Ruiz-Canela M., Galmes-Panades A.M., Abete I., Babio N., Fiol M., Martín-Sánchez V., Estruch R., Vidal J., Buil-Cosiales P. (2023). An Energy-Reduced Mediterranean Diet, Physical Activity, and Body Composition: An Interim Subgroup Analysis of the PREDIMED-Plus Randomized Clinical Trial. JAMA Netw. Open.

[184] Jennings A., Mulligan A.A., Khaw K.-T., Luben R.N., Welch A.A. (2020). A Mediterranean Diet Is Positively Associated with Bone and Muscle Health in a Non-Mediterranean Region in 25,450 Men and Women from EPIC-Norfolk. Nutrients.

[185] Jakše B., Pinter S., Jakše B., Bučar Pajek M., Pajek J. (2017). Effects of an Ad Libitum Consumed Low-Fat Plant-Based Diet Supplemented with Plant-Based Meal Replacements on Body Composition Indices. BioMed Res. Int..

[186] Wright N., Wilson L., Smith M., Duncan B., McHugh P. (2017). The BROAD study: A randomised controlled trial using a whole food plant-based diet in the community for obesity, ischaemic heart disease or diabetes. Nutr. Diabetes.

[187] Shan Z., Guo Y., Hu F.B., Liu L., Qi Q. (2020). Association of Low-Carbohydrate and Low-Fat Diets with Mortality Among US Adults. JAMA Intern. Med..

[188] Seidelmann S.B., Claggett B., Cheng S., Henglin M., Shah A., Steffen L.M., Folsom A.R., Rimm E.B., Willett W.C., Solomon S.D. (2018). Dietary carbohydrate intake and mortality: A prospective cohort study and meta-analysis. Lancet Public Health.

[189] Akter S., Mizoue T., Nanri A., Goto A., Noda M., Sawada N., Yamaji T., Iwasaki M., Inoue M., Tsugane S. (2021). Low carbohydrate diet and all cause and cause-specific mortality. Clin. Nutr..

[190] Volek J.S., Phinney S.D., Krauss R.M., Johnson R.J., Saslow L.R., Gower B., Yancy W.S., King J.C., Hecht F.M., Teicholz N. (2021). Alternative Dietary Patterns for Americans: Low-Carbohydrate Diets. Nutrients.

[191] Barnard N.D., Alwarith J., Rembert E., Brandon L., Nguyen M., Goergen A., Horne T., Nascimento G.F.D., Lakkadi K., Tura A. (2022). A Mediterranean Diet and Low-Fat Vegan Diet to Improve Body Weight and Cardiometabolic Risk Factors: A Randomized, Cross-over Trial. J. Am. Nutr. Assoc..

[192] Kahleova H., Petersen K.F., Shulman G.I., Alwarith J., Rembert E., Tura A., Hill M., Holubkov R., Barnard N.D. (2020). Effect of a Low-Fat Vegan Diet on Body Weight, Insulin Sensitivity, Postprandial Metabolism, and Intramyocellular and Hepatocellular Lipid Levels in Overweight Adults: A Randomized Clinical Trial. JAMA Netw. Open.

[193] Moon J., Koh G. (2020). Clinical Evidence and Mechanisms of High-Protein Diet-Induced Weight Loss. J. Obes. Metab. Syndr..

[194] Ai Y., Xu R., Liu L. (2021). The prevalence and risk factors of sarcopenia in patients with type 2 diabetes mellitus: A systematic review and meta-analysis. Diabetol. Metab. Syndr..

[195] Mesinovic J., Zengin A., De Courten B., Ebeling P.R., Scott D. (2019). Sarcopenia and type 2 diabetes mellitus: A bidirectional relationship. Diabetes Metab. Syndr. Obes..

[196] Basciani S., Camajani E., Contini S., Persichetti A., Risi R., Bertoldi L., Strigari L., Prossomariti G., Watanabe M., Mariani S. (2020). Very-Low-Calorie Ketogenic Diets With Whey, Vegetable, or Animal Protein in Patients With Obesity: A Randomized Pilot Study. J. Clin. Endocrinol. Metab..

[197] Castellana M., Conte E., Cignarelli A., Perrini S., Giustina A., Giovanella L., Giorgino F., Trimboli P. (2020). Efficacy and safety of very low calorie ketogenic diet (VLCKD) in patients with overweight and obesity: A systematic review and meta-analysis. Rev. Endocr. Metab. Disord..

[198] Lean M.E.J., Leslie W.S., Barnes A.C., Brosnahan N., Thom G., McCombie L., Peters C., Zhyzhneuskaya S., Al-Mrabeh A., Hollingsworth K.G. (2019). Durability of a primary care-led weight-management intervention for remission of type 2 diabetes: 2-year results of the DiRECT open-label, cluster-randomised trial. Lancet Diabetes Endocrinol..

[199] Juray S., Axen K.V., Trasino S.E. (2021). Remission of Type 2 Diabetes with Very Low-Calorie Diets-A Narrative Review. Nutrients.

[200] Manoogian E.N.C., Chow L.S., Taub P.R., Laferrère B., Panda S. (2022). Time-restricted Eating for the Prevention and Management of Metabolic Diseases. Endocr. Rev..

[201] Elortegui Pascual P., Rolands M.R., Eldridge A.L., Kassis A., Mainardi F., Lê K.-A., Karagounis L.G., Gut P., Varady K.A. (2023). A meta-analysis comparing the effectiveness of alternate day fasting, the 5:2 diet, and time-restricted eating for weight loss. Obesity.

[202] Laferrère B., Panda S. (2022). Calorie and Time Restriction in Weight Loss. N. Engl. J. Med..

[203] Attinà A., Leggeri C., Paroni R., Pivari F., Dei Cas M., Mingione A., Dri M., Marchetti M., Di Renzo L. (2021). Fasting: How to Guide. Nutrients.

[204] Corley B.T., Carroll R.W., Hall R.M., Weatherall M., Parry-Strong A., Krebs J.D. (2018). Intermittent fasting in Type 2 diabetes mellitus and the risk of hypoglycaemia: A randomized controlled trial. Diabet. Med. J. Br. Diabet. Assoc..

[205] Wang X., Li Q., Liu Y., Jiang H., Chen W. (2021). Intermittent fasting versus continuous energy-restricted diet for patients with type 2 diabetes mellitus and metabolic syndrome for glycemic control: A systematic review and meta-analysis of randomized controlled trials. Diabetes Res. Clin. Pract..

[206] Kelly R.K., Calhoun J., Hanus A., Payne-Foster P., Stout R., Sherman B.W. (2023). Increased dietary fiber is associated with weight loss among Full Plate Living program participants. Front. Nutr..

[207] Appel L.J., Moore T.J., Obarzanek E., Vollmer W.M., Svetkey L.P., Sacks F.M., Bray G.A., Vogt T.M., Cutler J.A., Windhauser M.M. (1997). A Clinical Trial of the Effects of Dietary Patterns on Blood Pressure. N. Engl. J. Med..

[208] Juul F., Vaidean G., Lin Y., Deierlein A.L., Parekh N. (2021). Ultra-Processed Foods and Incident Cardiovascular Disease in the Framingham Offspring Study. JACC.

[209] Dennis K.K., Wang F., Li Y., Manson J.E., Rimm E.B., Hu F.B., Willett W.C., Stampfer M.J., Wang D.D. (2023). Associations of dietary sugar types with coronary heart disease risk: A prospective cohort study. Am. J. Clin. Nutr..

[210] Mendoza K., Smith-Warner S.A., Rossato S.L., Khandpur N., Manson J.E., Qi L., Rimm E.B., Mukamal K.J., Willett W.C., Wang M. (2024). Ultra-processed foods and cardiovascular disease: Analysis of three large US prospective cohorts and a systematic review and meta-analysis of prospective cohort studies. Lancet Reg. Health–Am..

[211] Crosby L., Rembert E., Levin S., Green A., Ali Z., Jardine M., Nguyen M., Elliott P., Goldstein D., Freeman A. (2022). Changes in Food and Nutrient Intake and Diet Quality on a Low-Fat Vegan Diet Are Associated with Changes in Body Weight, Body Composition, and Insulin Sensitivity in Overweight Adults: A Randomized Clinical Trial. J. Acad. Nutr. Diet..

[212] Satija A., Malik V., Rimm E.B., Sacks F., Willett W., Hu F.B. (2019). Changes in intake of plant-based diets and weight change: Results from 3 prospective cohort studies. Am. J. Clin. Nutr..

[213] Neufingerl N., Eilander A. (2022). Nutrient Intake and Status in Adults Consuming Plant-Based Diets Compared to Meat-Eaters: A Systematic Review. Nutrients.

[214] Chacón V., Cara K.C., Chung M., Wallace T.C. (2025). Defining “low-carb” in the scientific literature: A scoping review of clinical studies. Crit. Rev. Food Sci. Nutr..

[215] Naude C.E., Brand A., Schoonees A., Nguyen K.A., Chaplin M., Volmink J. (2022). Low-carbohydrate versus balanced-carbohydrate diets for reducing weight and cardiovascular risk. Cochrane Database Syst. Rev..

[216] Churuangsuk C., Kherouf M., Combet E., Lean M. (2018). Low-carbohydrate diets for overweight and obesity: A systematic review of the systematic reviews. Obes. Rev..

[217] Chawla S., Tessarolo Silva F., Amaral Medeiros S., Mekary R.A., Radenkovic D. (2020). The effect of low-fat and low-carbohydrate diets on weight loss and lipid levels: A systematic review and meta-analysis. Nutrients.

[218] Kerna N.A., Ngwu D.C., Jomsky B.M., Holets H.M., Nnake I., Jeremiah S.M., Flores J.V., Pruitt K.D., Carsrud N.D.V., Senat A.J.B. (2024). Impact of Detox Diets on Obesity and Metabolic Syndrome: Implications for Weight Loss, Metabolic Health, and Clinical Practice. Eur. J. Med. Health Res..

[219] Jenkins D.J.A., Wong J.M.W., Kendall C.W.C., Esfahani A., Ng V.W.Y., Leong T.C.K., Faulkner D.A., Vidgen E., Paul G., Mukherjea R. (2014). Effect of a 6-month vegan low-carbohydrate (‘Eco-Atkins’) diet on cardiovascular risk factors and body weight in hyperlipidaemic adults: A randomised controlled trial. BMJ Open.

[220] Kovell L.C., Yeung E.H., Miller E.R., Appel L.J., Christenson R.H., Rebuck H., Schulman S.P., Juraschek S.P. (2020). Healthy diet reduces markers of cardiac injury and inflammation regardless of macronutrients: Results from the OmniHeart trial. Int. J. Cardiol..

[221] Ard J.D., Neeland I.J., Rothberg A.E., Chilton R.J., de Luis D., Cohen S.S., Johansen O.E. (2024). The OPTIFAST total and partial meal replacement programme reduces cardiometabolic risk in adults with obesity: Secondary and exploratory analysis of the OPTIWIN study. Diabetes Obes. Metab..

[222] Coleman C.D., Kiel J.R., Mitola A.H., Langford J.S., Davis K.N., Arterburn L.M. (2015). Effectiveness of a Medifast meal replacement program on weight, body composition and cardiometabolic risk factors in overweight and obese adults: A multicenter systematic retrospective chart review study. Nutr. J..

[223] Churuangsuk C., Hall J., Reynolds A., Griffin S.J., Combet E., Lean M.E.J. (2022). Diets for weight management in adults with type 2 diabetes: An umbrella review of published meta-analyses and systematic review of trials of diets for diabetes remission. Diabetologia.

[224] Liu D., Huang Y., Huang C., Yang S., Wei X., Zhang P., Guo D., Lin J., Xu B., Li C. (2022). Calorie Restriction with or without Time-Restricted Eating in Weight Loss. N. Engl. J. Med..

[225] Meessen E.C.E., Andresen H., van Barneveld T., van Riel A., Johansen E.I., Kolnes A.J., Kemper E.M., Olde Damink S.W.M., Schaap F.G., Romijn J.A. (2022). Differential Effects of One Meal per Day in the Evening on Metabolic Health and Physical Performance in Lean Individuals. Front. Physiol..

[226] Scholtens E.L., Krebs J.D., Corley B.T., Hall R.M. (2020). Intermittent fasting 5:2 diet: What is the macronutrient and micronutrient intake and composition?. Clin. Nutr..

[227] Xiao Y., Liu Y., Zhao L., Zhou Y. (2022). Effect of 5:2 Fasting Diet on Liver Fat Content in Patients with Type 2 Diabetic with Nonalcoholic Fatty Liver Disease. Metab. Syndr. Relat. Disord..

[228] Barski L., Eshkoli T., Brandstaetter E., Jotkowitz A. (2019). Euglycemic diabetic ketoacidosis. Eur. J. Intern. Med..

[229] Ezpeleta M., Cienfuegos S., Lin S., Pavlou V., Gabel K., Varady K.A. (2024). Efficacy and safety of prolonged water fasting: A narrative review of human trials. Nutr. Rev..

[230] van den Burg E.L., Schoonakker M.P., van Peet P.G., van den Akker-van Marle E.M., Lamb H.J., Longo V.D., Numans M.E., Pijl H. (2024). Integration of a fasting-mimicking diet programme in primary care for type 2 diabetes reduces the need for medication and improves glycaemic control: A 12-month randomised controlled trial. Diabetologia.

[231] Sadeghian M., Hosseini S.A., Zare Javid A., Ahmadi Angali K., Mashkournia A. (2021). Effect of Fasting-Mimicking Diet or Continuous Energy Restriction on Weight Loss, Body Composition, and Appetite-Regulating Hormones Among Metabolically Healthy Women with Obesity: A Randomized Controlled, Parallel Trial. Obes. Surg..

[232] Lobelo F., Rohm Young D., Sallis R., Garber M.D., Billinger S.A., Duperly J., Hutber A., Pate R.R., Thomas R.J., Widlansky M.E. (2018). Routine Assessment and Promotion of Physical Activity in Healthcare Settings: A Scientific Statement From the American Heart Association. Circulation.

[233] Swift D.L., McGee J.E., Earnest C.P., Carlisle E., Nygard M., Johannsen N.M. (2018). The Effects of Exercise and Physical Activity on Weight Loss and Maintenance. Prog. Cardiovasc. Dis..

[234] Niezgoda N., Chomiuk T., Kasiak P., Mamcarz A., Śliż D. (2025). The Impact of Physical Activity on Weight Loss in Relation to the Pillars of Lifestyle Medicine—A Narrative Review. Nutrients.

[235] US Department of Health and Human Services (2018). Physical Activity Guidelines for Americans.

[236] Eckel R.H., Jakicic J.M., Ard J.D., de Jesus J.M., Houston Miller N., Hubbard V.S., Lee I.-M., Lichtenstein A.H., Loria C.M., Millen B.E. (2014). 2013 AHA/ACC guideline on lifestyle management to reduce cardiovascular risk: A report of the American College of Cardiology/American Heart Association Task Force on Practice Guidelines. Circulation.

[237] Oppert J.-M., Ciangura C., Bellicha A. (2023). Physical activity and exercise for weight loss and maintenance in people living with obesity. Rev. Endocr. Metab. Disord..

[238] Oppert J.-M., Ciangura C., Bellicha A. (2025). Health-enhancing physical activity in obesity management: The need to (seriously) go beyond weight loss. Int. J. Obes..

[239] Johnson N.A., Sultana R.N., Brown W.J., Bauman A.E., Gill T. (2021). Physical activity in the management of obesity in adults: A position statement from Exercise and Sport Science Australia. J. Sci. Med. Sport.

[240] Funabashi D., Dobashi S., Sameshima K., Sagayama H., Nishijima T., Matsui T. (2024). Acute Vigorous Exercise Decreases Subsequent Nonexercise Physical Activity and Body Temperature Linked to Weight Gain. Med. Sci. Sports Exerc..

[241] Mandsager K., Harb S., Cremer P., Phelan D., Nissen S.E., Jaber W. (2018). Association of Cardiorespiratory Fitness with Long-term Mortality Among Adults Undergoing Exercise Treadmill Testing. JAMA Netw. Open.

[242] Fernández-Jiménez C., Dumitrache C.G., Rubio L., Ruiz-Montero P.J. (2024). Self-perceptions of ageing and perceived health status: The mediating role of cognitive functioning and physical activity. Ageing Soc..

[243] Dantas W.S., Roschel H., Murai I.H., Gil S., Davuluri G., Axelrod C.L., Ghosh S., Newman S.S., Zhang H., Shinjo S.K. (2020). Exercise-Induced Increases in Insulin Sensitivity After Bariatric Surgery Are Mediated by Muscle Extracellular Matrix Remodeling. Diabetes.

[244] Gil S., Peçanha T., Dantas W.S., Murai I.H., Merege-Filho C.A.A., de Sá-Pinto A.L., Pereira R.M.R., de Cleva R., Santo M.A., Rezende D.A.N. (2021). Exercise Enhances the Effect of Bariatric Surgery in Markers of Cardiac Autonomic Function. Obes. Surg..

[245] Kim H., Reece J., Kang M. (2020). Effects of Accumulated Short Bouts of Exercise on Weight and Obesity Indices in Adults: A Meta-Analysis. Am. J. Health Promot. AJHP.

[246] Sanders J.P., Biddle S.J.H., Gokal K., Sherar L.B., Skrybant M., Parretti H.M., Ives N., Yates T., Mutrie N., Daley A.J. (2021). ‘Snacktivity^TM^’ to increase physical activity: Time to try something different?. Prev. Med..

[247] Fyfe J.J., Hamilton D.L., Daly R.M. (2022). Minimal-Dose Resistance Training for Improving Muscle Mass, Strength, and Function: A Narrative Review of Current Evidence and Practical Considerations. Sports Med..

[248] Cruz-Jentoft A.J., Bahat G., Bauer J., Boirie Y., Bruyère O., Cederholm T., Cooper C., Landi F., Rolland Y., Sayer A.A. (2019). Sarcopenia: Revised European consensus on definition and diagnosis. Age Ageing.

[249] Dent E., Woo J., Scott D., Hoogendijk E.O. (2021). Toward the recognition and management of sarcopenia in routine clinical care. Nat. Aging.

[250] Conte C., Hall K.D., Klein S. (2024). Is Weight Loss–Induced Muscle Mass Loss Clinically Relevant?. JAMA.

[251] Azzolino D., Lucchi T. (2025). Expanding applications of GLP-1 therapies: A careful view. Int. J. Obes..

[252] Neeland I.J., Linge J., Birkenfeld A.L. (2024). Changes in lean body mass with glucagon-like peptide-1-based therapies and mitigation strategies. Diabetes Obes. Metab..

[253] Mendes C., Carvalho M., Bravo J., Martins S., Raimundo A. (2025). How Weight Loss After Bariatric Surgery Affects Sarcopenia Parameters and Diagnosis. Surgeries.

[254] Pekař M., Pekařová A., Bužga M., Holéczy P., Soltes M. (2020). The risk of sarcopenia 24 months after bariatric surgery—Assessment by dual energy X-ray absorptiometry (DEXA): A prospective study. Videosurgery Miniinvasive Tech..

[255] Prado C.M., Phillips S.M., Gonzalez M.C., Heymsfield S.B. (2024). Muscle matters: The effects of medically induced weight loss on skeletal muscle. Lancet Diabetes Endocrinol..

[256] Roh E., Choi K.M. (2020). Health Consequences of Sarcopenic Obesity: A Narrative Review. Front. Endocrinol..

[257] Morton R.W., McGlory C., Phillips S.M. (2015). Nutritional interventions to augment resistance training-induced skeletal muscle hypertrophy. Front. Physiol..

[258] Vikberg S., Sörlén N., Brandén L., Johansson J., Nordström A., Hult A., Nordström P. (2019). Effects of Resistance Training on Functional Strength and Muscle Mass in 70-Year-Old Individuals With Pre-sarcopenia: A Randomized Controlled Trial. J. Am. Med. Dir. Assoc..

[259] Hansen D., Decroix L., Devos Y., Nocca D., Cornelissen V., Dillemans B., Lannoo M. (2020). Towards Optimized Care After Bariatric Surgery by Physical Activity and Exercise Intervention: A Review. Obes. Surg..

[260] Vieira F.T., de Oliveira G.S., Gonçalves V.S.S., Neri S.G., de Carvalho K.M.B., Dutra E.S. (2022). Effect of physical exercise on muscle strength in adults following bariatric surgery: A systematic review and meta-analysis of different muscle strength assessment tests. PLoS ONE.

[261] Bellicha A., Van Baak M.A., Battista F., Beaulieu K., Blundell J.E., Busetto L., Carraça E.V., Dicker D., Encantado J., Ermolao A. (2021). Effect of exercise training before and after bariatric surgery: A systematic review and meta-analysis. Obes. Rev..

[262] Grier T., Brooks R.D., Solomon Z., Jones B.H. (2022). Injury Risk Factors Associated with Weight Training. J. Strength Cond. Res..

[263] Richmond S.A., Nettel-Aguirre A., Doyle-Baker P.K., Macpherson A., Emery C.A. (2016). Examining Measures of Weight as Risk Factors for Sport-Related Injury in Adolescents. J. Sports Med..

[264] Lauersen J.B., Andersen T.E., Andersen L.B. (2018). Strength training as superior, dose-dependent and safe prevention of acute and overuse sports injuries: A systematic review, qualitative analysis and meta-analysis. Br. J. Sports Med..

[265] Hanlon C., Krzak J.J., Prodoehl J., Hall K.D. (2020). Effect of Injury Prevention Programs on Lower Extremity Performance in Youth Athletes: A Systematic Review. Sports Health Multidiscip. Approach.

[266] Al Attar W.S.A., Ghulam H., Al Arifi S., Alomar A.I., Alhosaini S., Alharbi S., Alraddadi Y., Sanders R.H. (2023). Injury prevention programs including balance exercises with compliance and follow-up reduce the incidence of knee injuries in athletes: A systematic review and meta-analysis. Isokinet. Exerc. Sci..

[267] Pocovi N.C., De Campos T.F., Christine Lin C.-W., Merom D., Tiedemann A., Hancock M.J. (2022). Walking, Cycling, and Swimming for Nonspecific Low Back Pain: A Systematic Review with Meta-analysis. J. Orthop. Sports Phys. Ther..

[268] Alkatan M., Baker J.R., Machin D.R., Park W., Akkari A.S., Pasha E.P., Tanaka H. (2016). Improved Function and Reduced Pain after Swimming and Cycling Training in Patients with Osteoarthritis. J. Rheumatol..

[269] Flore G., Preti A., Carta M.G., Deledda A., Fosci M., Nardi A.E., Loviselli A., Velluzzi F. (2022). Weight Maintenance after Dietary Weight Loss: Systematic Review and Meta-Analysis on the Effectiveness of Behavioural Intensive Intervention. Nutrients.

[270] Hall K.D., Kahan S. (2018). Maintenance of Lost Weight and Long-Term Management of Obesity. Med. Clin..

[271] Martínez-Gómez M.G., Roberts B.M. (2022). Metabolic adaptations to weight loss: A brief review. J. Strength Cond. Res..

[272] Mu W.-J., Zhu J.-Y., Chen M., Guo L. (2021). Exercise-mediated browning of white adipose tissue: Its significance, mechanism and effectiveness. Int. J. Mol. Sci..

[273] Dinas P.C., Lahart I.M., Timmons J.A., Svensson P.-A., Koutedakis Y., Flouris A.D., Metsios G.S. (2017). Effects of physical activity on the link between PGC-1a and FNDC5 in muscle, circulating Irisin and UCP1 of white adipocytes in humans: A systematic review. F1000Research.

[274] Cleven L., Syrjanen J.A., Geda Y.E., Christenson L.R., Petersen R.C., Vassilaki M., Woll A., Krell-Roesch J. (2023). Association between physical activity and longitudinal change in body mass index in middle-aged and older adults. BMC Public Health.

[275] Cao X., Thyfault J.P. (2023). Exercise drives metabolic integration between muscle, adipose and liver metabolism and protects against aging-related diseases. Exp. Gerontol..

[276] Grevendonk L., Connell N.J., McCrum C., Fealy C.E., Bilet L., Bruls Y.M.H., Mevenkamp J., Schrauwen-Hinderling V.B., Jörgensen J.A., Moonen-Kornips E. (2021). Impact of aging and exercise on skeletal muscle mitochondrial capacity, energy metabolism, and physical function. Nat. Commun..

[277] Wang Z., Ying Z., Bosy-Westphal A., Zhang J., Heller M., Later W., Heymsfield S.B., Müller M.J. (2011). Evaluation of specific metabolic rates of major organs and tissues: Comparison between men and women. Am. J. Hum. Biol. Off. J. Hum. Biol. Counc..

[278] Ashtary-Larky D., Bagheri R., Abbasnezhad A., Tinsley G.M., Alipour M., Wong A. (2020). Effects of gradual weight loss v. rapid weight loss on body composition and RMR: A systematic review and meta-analysis. Br. J. Nutr..

[279] Petridou A., Siopi A., Mougios V. (2019). Exercise in the management of obesity. Metabolism.

[280] Paixão C., Dias C.M., Jorge R., Carraça E.V., Yannakoulia M., de Zwaan M., Soini S., Hill J.O., Teixeira P.J., Santos I. (2020). Successful weight loss maintenance: A systematic review of weight control registries. Obes. Rev..

[281] Melby C.L., Paris H.L., Sayer R.D., Bell C., Hill J.O. (2019). Increasing energy flux to maintain diet-induced weight loss. Nutrients.

[282] Chung N., Park M.-Y., Kim J., Park H.-Y., Hwang H., Lee C.-H., Han J.-S., So J., Park J., Lim K. (2018). Non-exercise activity thermogenesis (NEAT): A component of total daily energy expenditure. J. Exerc. Nutr. Biochem..

[283] Perez L.C., Perez L.T., Nene Y., Umpierrez G.E., Davis G.M., Pasquel F.J. (2022). Interventions associated with brown adipose tissue activation and the impact on energy expenditure and weight loss: A systematic review. Front. Endocrinol..

[284] CDCMMWR (2024). QuickStats: Percentage of Adults Aged ≥25 Years Who Met the 2018 Federal Physical Activity Guidelines for Both Muscle-Strengthening and Aerobic Physical Activity, by Educational Attainment—United States, 2022. MMWR Morb. Mortal. Wkly. Rep..

[285] Vemulapalli S., Dolor R.J., Hasselblad V., Schmit K., Banks A., Heidenfelder B., Patel M.R., Jones W.S. (2015). Supervised vs unsupervised exercise for intermittent claudication: A systematic review and meta-analysis. Am. Heart J..

[286] Roy M., Williams S.M., Brown R.C., Meredith-Jones K.A., Osborne H., Jospe M., Taylor R.W. (2018). High-Intensity Interval Training in the Real World: Outcomes from a 12-Month Intervention in Overweight Adults. Med. Sci. Sports Exerc..

[287] Bannell D.J., France-Ratcliffe M., Buckley B.J.R., Crozier A., Davies A.P., Hesketh K.L., Jones H., Cocks M., Sprung V.S., on behalf of the MOTIVATE Team (2023). Adherence to unsupervised exercise in sedentary individuals: A randomised feasibility trial of two mobile health interventions. Digit. Health.

[288] Lacroix A., Hortobágyi T., Beurskens R., Granacher U. (2017). Effects of Supervised vs. Unsupervised Training Programs on Balance and Muscle Strength in Older Adults: A Systematic Review and Meta-Analysis. Sports Med..

[289] Gabay M., Levi O., Petracovschi S., Negrea C., Matichescu M., Oravitan M. (2023). Exploring exercise adherence and quality of life among veteran, novice, and dropout trainees. Front. Sports Act. Living.

[290] Shibuya K., Ji X., Pfoh E.R., Milinovich A., Weng W., Bauman J., Ganguly R., Misra-Hebert A.D., Hobbs T.M., Kattan M.W. (2020). Association between shared medical appointments and weight loss outcomes and anti-obesity medication use in patients with obesity. Obes. Sci. Pract..

[291] Kirbach K., Marshall-Moreno I., Shen A., Cullen C., Sanigepalli S., Bobadilla A., MacElhern L., Grunvald E., Kallenberg G., Tristão Parra M. (2024). Implementation of a virtual, shared medical appointment program that focuses on food as medicine principles in a population with obesity: The SLIM program. Front. Nutr..

[292] Wadsworth K.H., Archibald T.G., Payne A.E., Cleary A.K., Haney B.L., Hoverman A.S. (2019). Shared medical appointments and patient-centered experience: A mixed-methods systematic review. BMC Fam. Pract..

[293] Walker R., Ramasamy V., Sturgiss E., Dunbar J., Boyle J. (2022). Shared medical appointments for weight loss: A systematic review. Fam. Pract..

[294] Yager S., Parker M., Luxenburg J., Varghai N.H. (2020). Evaluation of multidisciplinary weight loss shared medical appointments. J. Am. Pharm. Assoc..

[295] American Academy of Family Physicians Shared Medical Appointments/Group Visits. https://www.aafp.org/about/policies/all/shared-medical-appointments.html.

[296] Center for Medicare and Medicaid Services National Coverage Determination—Medical Nutrition Therapy (180.1). Medicare Coverage Database. https://www.cms.gov/medicare-coverage-database/view/ncd.aspx?ncdid=252.

[297] Brown J., Clarke C., Stoklossa C., Sievenpiper J. (2022). Medical Nutrition Therapy in Obesity Management.

[298] Medicare Learning Network Telehealth and Remote Patient Monitoring—Medicare Learning Network. Centers for Medicare and Medicaid Services. January 2025. https://www.cms.gov/files/document/mln901705-telehealth-remote-patient-monitoring.pdf.

[299] de Farias F.A.C., Dagostini C.M., Bicca Y.d.A., Falavigna V.F., Falavigna A. (2020). Remote Patient Monitoring: A Systematic Review. Telemed. E-Health.

[300] Noah B., Keller M.S., Mosadeghi S., Stein L., Johl S., Delshad S., Tashjian V.C., Lew D., Kwan J.T., Jusufagic A. (2018). Impact of remote patient monitoring on clinical outcomes: An updated meta-analysis of randomized controlled trials. Npj Digit. Med..

[301] Medicare Learning Network Medicare Learning Network—Chronic Care Management Services. Centers for Medicare and Medicaid Services. May 2024. https://www.cms.gov/outreach-and-education/medicare-learning-network-mln/mlnproducts/downloads/chroniccaremanagement.pdf.

[302] Basu S., Phillips R.S., Bitton A., Song Z., Landon B.E. (2015). Medicare Chronic Care Management Payments and Financial Returns to Primary Care Practices: A Modeling Study. Ann. Intern. Med..

[303] O’Malley A.S., Sarwar R., Keith R., Balke P., Ma S., McCall N. (2017). Provider Experiences with Chronic Care Management (CCM) Services and Fees: A Qualitative Research Study. J. Gen. Intern. Med..

[304] Jacobs M., Harris J., Craven K., Sastre L. (2021). Sharing the “weight” of obesity management in primary care: Integration of registered dietitian nutritionists to provide intensive behavioural therapy for obesity for Medicare patients. Fam. Pract..

[305] Luo Z., Gritz M., Connelly L., Dolor R.J., Phimphasone-Brady P., Li H., Fitzpatrick L., Gales M., Shah N., Holtrop J.S. (2021). A Survey of Primary Care Practices on Their Use of the Intensive Behavioral Therapy for Obese Medicare Patients. J. Gen. Intern. Med..

[306] Tapsell L.C., Neale E.P. (2016). The Effect of Interdisciplinary Interventions on Risk Factors for Lifestyle Disease: A Literature Review. Health Educ. Behav..

[307] Clinical Exercise Physiology Association What is Clinical Exercise Physiology? Clinical Exercise Physiology Association—An ACSM Affiliate Society. https://www.acsm-cepa.org/content.aspx?page_id=22&club_id=324409&module_id=291959.

[308] Magal M., Neric F.B. (2020). ACSM Certifications: Defining an Exercise Profession from Concept to Assessment. ACSMs Health Fit. J..

[309] Boehmer K.R., Barakat S., Ahn S., Prokop L.J., Erwin P.J., Murad M.H. (2016). Health coaching interventions for persons with chronic conditions: A systematic review and meta-analysis protocol. Syst. Rev..

[310] Silberman J.M., Kaur M., Sletteland J., Venkatesan A. (2020). Outcomes in a digital weight management intervention with one-on-one health coaching. PLoS ONE.

[311] Chew H.S.J., Rajasegaran N.N., Chin Y.H., Chew W.S.N., Kim K.M. (2023). Effectiveness of Combined Health Coaching and Self-Monitoring Apps on Weight-Related Outcomes in People With Overweight and Obesity: Systematic Review and Meta-analysis. J. Med. Internet Res..

[312] Hesseldal L., Christensen J.R., Olesen T.B., Olsen M.H., Jakobsen P.R., Laursen D.H., Lauridsen J.T., Nielsen J.B., Søndergaard J., Brandt C.J. (2022). Long-term Weight Loss in a Primary Care-Anchored eHealth Lifestyle Coaching Program: Randomized Controlled Trial. J. Med. Internet Res..

[313] Sieczkowska S.M., de Lima A.P., Swinton P.A., Dolan E., Roschel H., Gualano B. (2021). Health Coaching Strategies for Weight Loss: A Systematic Review and Meta-Analysis. Adv. Nutr..

[314] Jay M.R., Wittleder S., Vandyousefi S., Illenberger N., Nicholson A., Sweat V., Meissner P., Angelotti G., Ruan A., Wong L. (2024). A Cluster-Randomized Study of Technology-Assisted Health Coaching for Weight Management in Primary Care. Ann. Fam. Med..

[315] Halley M.C., Petersen J., Nasrallah C., Szwerinski N., Romanelli R., Azar K.M. (2020). Barriers and Facilitators to Real-world Implementation of the Diabetes Prevention Program in Large Healthcare Systems: Lifestyle Coach Perspectives. J. Gen. Intern. Med..

[316] An S., Song R. (2020). Effects of health coaching on behavioral modification among adults with cardiovascular risk factors: Systematic review and meta-analysis. Patient Educ. Couns..

[317] Nielsen S.S., Christensen J.R. (2018). Occupational Therapy for Adults with Overweight and Obesity: Mapping Interventions Involving Occupational Therapists. Occup. Ther. Int..

[318] Macchi M., Spezia M., Elli S., Schiaffini G., Chisari E. (2020). Obesity Increases the Risk of Tendinopathy, Tendon Tear and Rupture, and Postoperative Complications: A Systematic Review of Clinical Studies. Clin. Orthop..

[319] Lynch D.H., Petersen C.L., Fanous M.M., Spangler H.B., Kahkoska A.R., Jimenez D., Batsis J.A. (2022). The relationship between multimorbidity, obesity and functional impairment in older adults. J. Am. Geriatr. Soc..

[320] IQWiG (2024). In brief: Physical therapy. InformedHealth.org [Internet].

[321] Mechanick J.I., Mechanick J.I., Kushner R.F. (2025). Pharmacotherapy De-Escalation as a Critical Component of Lifestyle Medicine. Lifestyle Medicine: Closing Research, Practice, and Knowledge Gaps.

[322] Valliant S.N., Burbage S.C., Pathak S., Urick B.Y. (2022). Pharmacists as accessible health care providers: Quantifying the opportunity. J. Manag. Care Spec. Pharm..

[323] Burns R.B., Jay M.R., Thorndike A.N., Kanjee Z. (2024). How Would You Manage This Patient With Obesity? Grand Rounds Discussion From Beth Israel Deaconess Medical Center. Ann. Intern. Med..

[324] Moiz A., Filion K.B., Toutounchi H., Tsoukas M.A., Yu O.H.Y., Peters T.M., Eisenberg M.J. (2025). Efficacy and Safety of Glucagon-Like Peptide-1 Receptor Agonists for Weight Loss Among Adults Without Diabetes. Ann. Intern. Med..

[325] Christensen S., Robinson K., Thomas S., Williams D.R. (2024). Dietary intake by patients taking GLP-1 and dual GIP/GLP-1 receptor agonists: A narrative review and discussion of research needs. Obes. Pillars.

